# Catalytic ozone decomposition and adsorptive VOCs removal in bimetallic metal-organic frameworks

**DOI:** 10.1038/s41467-022-32678-2

**Published:** 2022-08-25

**Authors:** Chen Dong, Jia-Jia Yang, Lin-Hua Xie, Ganglong Cui, Wei-Hai Fang, Jian-Rong Li

**Affiliations:** 1 Beijing Key Laboratory for Green Catalysis and Separation, and Department of Environmental Chemical Engineering, Beijing University of Technology, 100124 Beijing, China; 2 Key Laboratory of Theoretical and Computational Photochemistry, Ministry of Education, College of Chemistry, Beijing Normal University, 100875 Beijing, China

**Keywords:** Pollution remediation, Metal-organic frameworks, Heterogeneous catalysis

## Abstract

Atmospheric ozone has long been a threat to human health, however, rational design of high-performance O_3_-decomposition catalysts remains challenging. Herein, we demonstrate the great potential of a series of isomorphous bimetallic MOFs denoted as PCN-250(Fe_2_M) (M = Co^2+^, Ni^2+^, Mn^2+^) in catalytic O_3_ decomposition. Particularly, PCN-250(Fe_2_Co) showed 100% O_3_ removal efficiency for a continuous air flow containing 1 ppm O_3_ over a wide humidity range (0 ‒ 80% RH) at room temperature. Mechanism studies suggested that the high catalytic performance originated from the introduction of open Co(II) sites as well as its porous structure. Additionally, at low pressures around 10 Pa, PCN-250(Fe_2_Co) exhibited high adsorption capacities (89 ‒ 241 mg g^−1^) for most VOCs, which are not only a class of hazardous air pollutants but also the precursor of O_3_. This work opens up a new avenue to develop advanced air purification materials for O_3_ and VOCs removal in one.

## Abstract (web summary)

Warm-season O_3_ pollution has been increasingly frequent worldwide in the past few years, exposing a threat to human health as well as the natural environment. Here, the authors showcase a stable MOF which can not only effectively capture various airborne VOCs, but decompose trace O_3_ in ambient air.

## Introduction

Ground-level ozone (O_3_) has long been a threat to human health, especially in urban area^1–3^. Outdoor O_3_ is mostly generated from the photochemical reactions between nitrogen dioxides and volatile organic compounds (VOCs) in the air under sunlight^4,5^. Indoor O_3_ is from indoor-outdoor air exchange as well as some emission sources include photocopiers, laser printers, ultraviolet lamps, and O_3_ disinfectors^6–8^. Although being highly reactive as a very strong oxidizing agent, O_3_ itself is relatively stable and nearly does not spontaneously decompose to oxygen (O_2_) at the concentrations (ppb level) that are typically encountered in ambient air^6^. Plenty of studies have demonstrated the potential risk of respiratory and cardiovascular mortality for the human body after long-term exposure to the air with a trace of O_3_^9,10^. The World Health Organization has recommended that the 8-h daily maximum O_3_ concentration in ambient air exceeding 100 μg m^−3^ (∼51 ppb) poses health risks^11^. However, the occurrence of severe warm-season O_3_ pollution has become more frequent in the past few years for both developing and developed countries^7,12^. For example, field observations and satellite retrievals reveal that the observed hourly maximum O_3_ concentrations in China frequently exceeded 150 ppb (severe O_3_ pollution)^13,14^. The study to reduce O_3_ pollution is thus in high demand for the protection of the environment and human health.

The existing approaches to remove O_3_ from ambient air are typically based on activated carbon adsorption, chemical absorption, and catalytic decomposition methods^15–18^. Among them, catalytic decomposition of O_3_ into O_2_ at low temperatures is promising because of its ease in operation, high efficiency, good persistence, and environmental friendliness^19,20^. Over the past decades, significant advances have been made in catalytic O_3_ degradation using transition metal (Mn^21^, Fe^22^, Co^23^, Ni^24^, and Cu^25^) oxides mostly. As a class of newly emerged materials, metal-organic frameworks (MOFs) have attracted great interest in various catalytic reactions because of their tunable structure and diverse functionality, including O_3_ catalytic decomposition^26–31^. In addition, compared with transition metal oxides, MOFs can have highly porous structures, thus may offer extra catalytically active sites on interior pore surface, and even serve as advanced multifunctional materials to decompose O_3_ and remove other airborne hazardous molecules by adsorption simultaneously^32–36^. Indeed, there exist kinds of air pollutants in some real application scenarios. For example, non-thermal plasma (NTP) is a highly efficient technology for VOCs removal and has been widely used for the past two decades, but the exhausted gases of NTP reactors usually contain the by-product O_3_ as well as some incompletely decomposed VOCs^37^. It is thus highly desirable but still challenging to develop highly efficient, porous, and stable MOFs as such multifunctional air filtering materials.

**Fig. 1:**
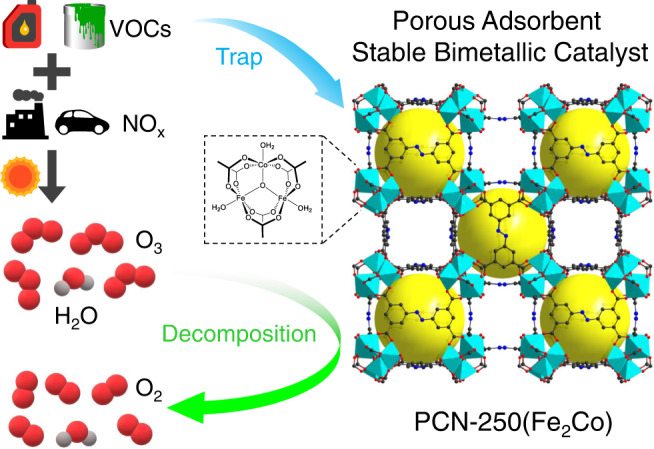
Schematic representation of the bimetallic MOF PCN-250(Fe_2_Co) as a porous catalyst for both O_3_ and VOCs removal. The O, C, N atoms, MO_6_ units (M = Fe or Co) and cages in PCN-250(Fe_2_Co) are represented by red, black, blue spheres, turquoise polyhedra, and large yellow spheres, respectively.

The catalytically active sites in MOFs are commonly metal centers, and most reported MOFs are monometallic. Generating multimetallic building units in MOFs has been recently proposed as an effective strategy to regulate their catalytic properties^38,39^. Exploring multimetallic MOFs for catalytic O_3_ decomposition would be intriguing. The MOF PCN-250(Fe_3_)^40^ (also known as MIL-127(Fe)^41^) constructed from Fe_3_-µ_3_-oxo clusters and ABTC^4−^ ligands (H_4_ABTC = 3,3′,5,5′-azobenzenetetracarboxylic acid) is a preferred platform for such a study because one Fe(III) ion of the Fe_3_-µ_3_-oxo cluster can be readily substituted by a common divalent transition metal ion, e.g., Co(II), Ni(II), and Mn(II), and this MOF can be facilely prepared with high stability and high porosity. Herein, we demonstrate the great potential of these bimetallic MOFs PCN-250(Fe_2_M) (M = Co^2+^, Ni^2+^, Mn^2+^) for catalytic O_3_ removal from ambient air, especially PCN-250(Fe_2_Co). It is also found that PCN-250(Fe_2_Co) shows a high capacity in the adsorptive removal of volatile organic compounds (VOCs) (Fig. 1), which are not only a class of hazardous air pollutants but also the precursor of O_3_. It is an attribution not available for most of the other O_3_-decomposition catalysts. This study suggests the great potential of MOF-based materials for O_3_ pollution control and also offers insights into the design of new air purification materials.

## Results

### Synthesis and characterization

Three bimetallic MOFs PCN-250(Fe_2_M) (M = Co^2+^, Ni^2+^, Mn^2+^), as well as their monometallic counterpart PCN-250(Fe_3_), were prepared by the reported method with some modifications to evaluate their performances on catalytic O_3_ decomposition. PXRD patterns of the four samples all showed strong peaks and well matched the simulated PXRD pattern of PCN-250(Fe_3_) (Supplementary Fig. 2), confirming that their structures are isomorphous and the samples are in high crystallinity and purity. N_2_ sorption isotherms of the four MOFs recorded at 77 K were all classic type-I isotherms for microporous materials (Supplementary Figs. 3–6). The pore volumes and BET surface areas of PCN-250(Fe_3_), PCN-250(Fe_2_Co), PCN-250(Fe_2_Ni), and PCN-250(Fe_2_Mn) were estimated to be 0.52, 0.59, 0.58, and 0.57 cm^3^ g^−1^, and 1408, 1478, 1465, and 1443 m^2^ g^−1^ with a pore size distribution of 7.0–11.5 Å, respectively. Thermal gravimetric analysis (TGA) curves suggested that the four MOFs thermally decomposed at about 400 °C (Supplementary Fig. 7). X-ray photoelectron spectra (XPS) analyses confirmed the presence of only Fe(III) ions in PCN-250(Fe_3_) and the presence of both divalent metal ions (Co^2+^, Ni^2+^, or Mn^2+^) and trivalent Fe^3+^ ions in the three bimetallic MOFs (Supplementary Figs. 8–11), which was further confirmed by the results of inductively coupled plasma-atomic emission spectroscopy (ICP-AES) measurements for digested samples of the MOFs (Supplementary Table 7) and EDS mapping images for the PCN-250 crystals (Supplementary Fig. 12).

### Catalytic activity for O_3_ decomposition

**Fig. 2:**
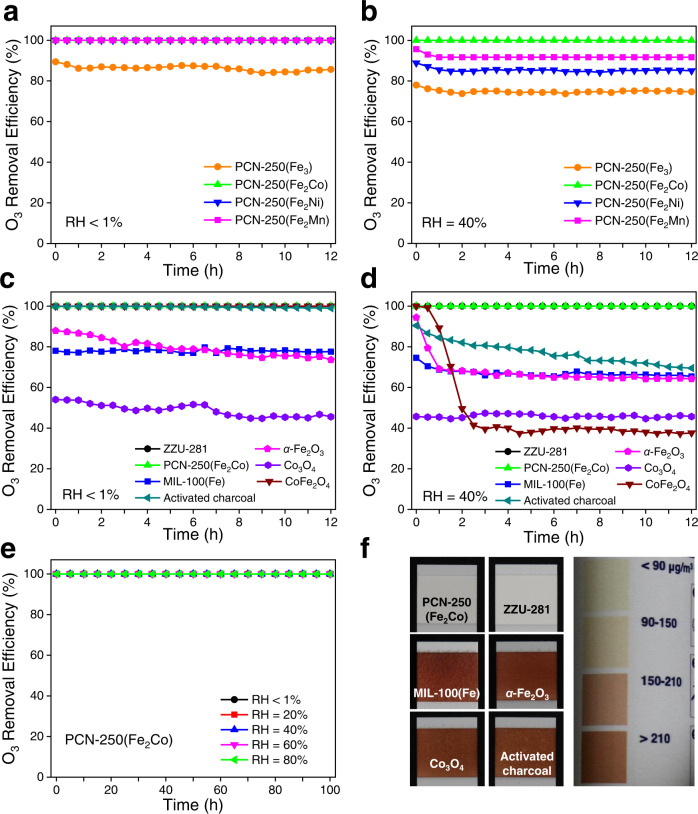
The O_3_ removal efficiencies for PCN-250 and other reference materials. **a** RH < 1% (zero air) and **b** RH = 40% for the PCN-250 samples. **c** RH < 1% (zero air) and **d** RH = 40% for PCN-250(Fe_2_Co), ZZU-281, MIL-100(Fe), *α*-Fe_2_O_3_, Co_3_O_4_, CoFe_2_O_4_, and activated charcoal. **e** Varied RHs for PCN-250(Fe_2_Co). Other conditions for all the above tests: 0.03 g catalyst diluted with 0.2 g quartz sand, concentration of O_3_ = 1 ppm, flow rate = 1 L min^−1^, room temperature. **f** O_3_ test strips used in the tests under humid condition (RH = 40%) and the color scale.

Catalytic O_3_ decomposition experiments were conducted with continuous-flow fixed-bed reactors of the tested materials (30 mg) at ambient temperature and pressure, and the inlet gas flow rate was 1 L min^−1^ (see supplementary information for details). Considering the concentration of O_3_ in ambient air is normally in the range of tens to hundreds of ppb, the O_3_ concentration of inlet gas was set to be 1 ppm (balance air). For dry inlet gas (zero air), the three bimetallic MOFs PCN-250(Fe_2_Co), PCN-250(Fe_2_Mn), and PCN-250(Fe_2_Ni) completely removed the O_3_ of inlet gas during 12 h of continuous testing, while the O_3_ removal efficiency of PCN-250(Fe_3_) was around 85% (Fig. 2a). When the humidity of the inlet gas increased to RH = 40% (relative humidity at room temperature), the performances of the four MOFs became more different (Fig. 2b). For PCN-250(Fe_2_Mn), PCN-250(Fe_2_Ni), and PCN-250(Fe_3_), the initial O_3_ removal efficiencies were 97%, 89%, and 78%, which gradually decreased in the first 2 h and stabilized at 92%, 85%, and 75% in the following 10 h, respectively. In contrast, PCN-250(Fe_2_Co) still retained 100% O_3_ removal efficiency throughout the test. These results revealed that the three bimetallic MOFs were more catalytically active than PCN-250(Fe_3_) for the decomposition reaction of O_3_, and the O_3_ removal performances of PCN-250(Fe_3_), PCN-250(Fe_2_Ni), and PCN-250(Fe_2_Mn) could be affected by the humidity of inlet gas. It is common that the O_3_ decomposition performances of catalysts reduce under more humid conditions^21,22^. Notably, PCN-250(Fe_2_Co) showed 100% O_3_ removal efficiency under both dry and humid conditions, suggesting that it has the highest catalytic activity among the four MOFs.

For comparison, the O_3_ removal performances of some representative materials were also evaluated under the same experimental conditions, including two MOFs previously reported to show high O_3_ degradation activities, ZZU-281^31^ and MIL-100(Fe)^30^, three metal oxides, *α*-Fe_2_O_3_^15^, Co_3_O_4_^22^, and CoFe_2_O_4_^42^, and a common adsorbent widely used in air purification, activated charcoal^43^. The structure, purity, and/or porosity of these reference materials were confirmed (Supplementary Figs. 16–22). As shown in Fig. 2c, d, the O_3_ removal efficiency of Co_3_O_4_ was around 45% under both dry and humid conditions. The O_3_ removal efficiencies of MIL-100(Fe) and *α*-Fe_2_O_3_ were close after 12 h continuous testing, being ∼75% and 65% under dry and humid conditions, respectively. The O_3_ removal efficiencies of activated charcoal and CoFe_2_O_4_ decreased from 91 to 70% and 100 to 40% within 12 h under humid conditions, respectively, although their O_3_ removal efficiencies under dry conditions were >99% throughout the test. The continuous decline in O_3_ removal performance of activated charcoal under both dry and humid conditions may be attributed to the gradual consumption of O_3_ reactive sites (e.g., hydroxyl groups) on the sample surface^44^. In addition, it is noteworthy that the O_3_ removal performance of the bimetallic oxide CoFe_2_O_4_ is quite different from that of the physical mixture of Co_3_O_4_ and *α-*Fe_2_O_3_ (Supplementary Fig. 23). Among these reference materials, only ZZU-281 completely removed O_3_ in inlet gases under both dry and humid conditions as PCN-250(Fe_2_Co) did. To further validate the experimental results, commercial O_3_ test strips were used to detect the O_3_ concentration of outlet gases that respectively passed through the materials under humid conditions (RH = 40%). As the O_3_ concentration of test gas increases, the strips change from white (ozone-free) to light-yellow (O_3_ concentrations around 90 µg m^−3^) and then to brown (O_3_ concentrations higher than 210 µg m^−3^) (Fig. 2f, right). The observed colors of the strips applied to the materials confirmed their different O_3_ removal efficiencies (Fig. 2f, left).

The O_3_ removal performances of PCN-250(Fe_2_Co) under various humidity and longer testing duration were also investigated. As shown in Fig. 2e, PCN-250(Fe_2_Co) kept 100% O_3_ removal efficiency within 100 h of continuous testing when the RH of inlet gas varied from <1% (dry zero air) to 80%. We further explored the effects of the weight amount of PCN-250(Fe_2_Co) and the O_3_ concentration of inlet gas on the O_3_ removal efficiency of PCN-250(Fe_2_Co). Control experiments were carried out after the weight amount of PCN-250(Fe_2_Co) was reduced from 30 mg to 15 mg or the O_3_ concentration of inlet gas was increased from 1 ppm to 50 ppm. For dry inlet gas, the O_3_ removal efficiency of PCN-250(Fe_2_Co) maintained 100% in both cases; for humid inlet gas (RH = 40% at room temperature), the O_3_ removal efficiency of PCN-250(Fe_2_Co) reduced to around 65% and 35%, respectively (Supplementary Fig. 24). These results revealed that the catalytic O_3_ degradation capacity of PCN-250(Fe_2_Co) under dry condition was higher than that under humid condition, as also observed for the other three PCN-250 MOFs. The different O_3_ removal efficiencies of PCN-250(Fe_2_Co) for dry and humid gases were also confirmed by cycling tests where the humidity of the gas flow was alternately changed (Supplementary Fig. 25). Under the dry condition, the O_3_ removal efficiency of PCN-250(Fe_2_Co) was 100% even if the O_3_ concentration of inlet gas was further increased to 200 ppm, even much higher than that of ZZU-281 under the same condition (Supplementary Fig. 26). The different O_3_ degradation performance of PCN-250(Fe_2_Co) under dry and humid conditions may be originated from two aspects. On the one hand, PCN-250(Fe_2_Co) is moderately hydrophilic as indicated by its water vapor adsorption isotherm at 298 K, where the water uptake gradually increases to 27 mmol g^−1^ at 0.6 *P*/*P*_0_ and keeps nearly unchanged at higher pressures (Supplementary Fig. 27). The water contact angle of the powder sample of PCN-250(Fe_2_Co) is about 11° (Supplementary Fig. 28), indicating that the crystal surface is also moderately hydrophilic. Under humid conditions, adsorbed water molecules would partially or even fully block the pores of PCN-250(Fe_2_Co), reducing the amount of accessible catalytically active sites on the interior pore surface to contact with O_3_ molecules. On the other hand, water molecules may participate in the catalytic O_3_ decomposition reaction, and the resulting new reaction pathway requires higher activation energy. Indeed, it was found that the O_3_ removal efficiency of PCN-250(Fe_2_Co) under humid conditions could be largely improved by generally heating the reactor to 45 °C (Supplementary Fig. 29).

**Fig. 3:**
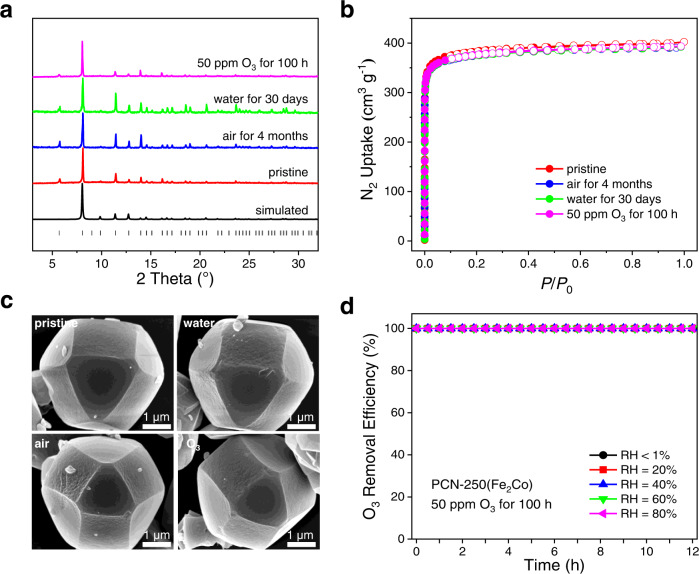
Stability tests for PCN-250(Fe_2_Co). **a** PXRD patterns, **b** N_2_ sorption isotherms, and **c** SEM images of the pristine and stability-tested PCN-250(Fe_2_Co) samples. **d** O_3_ removal efficiencies for PCN-250(Fe_2_Co) after 50 ppm O_3_ treatment. Test conditions: 0.03 g catalyst diluted with 0.2 g quartz sand, concentration of O_3_ = 1 ppm, flow rate = 1 L min^−1^, room temperature.

For the practical application of O_3_ removal, the long-term stability of catalysts under real working conditions is highly important^45^. The stability issue is especially outstanding for MOF-type catalysts since a large number of MOFs have been proven unstable even in ambient air^46^. In the last few years, a few MOFs showing high stability in water, even under harsh acidic or basic conditions, have been reported, whereas there are very little knowledge about the stability of MOFs when exposed to O_3_-containing air^47–52^. The high hydrolytic stability of PCN-250 MOFs has been well documented in the literature^53^. The long-term hydrolytic stability of PCN-250(Fe_2_Co) was further tested by soaking it in water at room temperature for 30 days and by exposing it to ambient air for 4 months. PXRD and N_2_ sorption measurement results revealed that the PCN-250(Fe_2_Co) samples well maintained their high crystallinity and porosity after these treatments (Fig. 3a, b). No obvious change was observed by SEM for the crystal morphology and surface of PCN-250(Fe_2_Co) (Fig. 3c).

As a strong oxidizing agent, O_3_ is highly reactive to many substances, e.g., destroying organic compounds containing carbon-carbon double bonds, carbon-carbon triple bonds, and porphyrin rings^54–56^. Therefore, besides hydrolytic stability, the materials used for O_3_ removal should also be able to resist long-term O_3_ attack. The tolerance of PCN-250(Fe_2_Co) to airborne O_3_ (1 ppm) was indicated by its stable O_3_ removal efficiency in the 100 h tests discussed above (Fig. 2e). Its tolerance was further evaluated by exposing it to a continuous flow (flow rate: 0.5 L min^−1^) of dry or humid air (RH = 40% at room temperature) containing 50 ppm O_3_ for 100 h (50 h for wet gas, and 50 h for dry gas) at room temperature. The comparison of PXRD patterns, N_2_ adsorption isotherms, and SEM images for the O_3_ treated and pristine PCN-250(Fe_2_Co) samples suggested a high tolerance of PCN-250(Fe_2_Co) to O_3_ containing ambient air (Fig. 3a–c). Further experiments indicated that the four PCN-250 samples all are highly tolerant of O_3_ (Supplementary Figs. 2–11). Moreover, the 50 ppm O_3_ treated PCN-250(Fe_2_Co) still showed 100% O_3_ removal efficiency to dry or humid air (RH = 40% at room temperature) containing 1 ppm O_3_ as the pristine sample did (Fig. 3d). Unexpectedly, it was found that some MOFs commonly regarded to be highly stable easily degraded after exposure to O_3_ containing ambient air, e.g., ZIF-8, ZIF-L, and MIL-101(Cr). After being exposed to the flow of humid air containing 1 ppm O_3_ for 24 h, the sample of ZIF-8 changed from white powder to light-yellow viscous liquid and completely lost its initial crystalline structure (Supplementary Fig. 30). The change was faster (in 4 h) when the O_3_ concentration was increased to 50 ppm. Similar results were also observed for ZIF-L, which is built from the same metal ions and organic ligands as ZIF-8 (Supplementary Fig. 31). Although being more tolerant of O_3_ in humid air than the two ZIFs, MIL-101(Cr) also lost crystallinity and changed color after exposing to the flow of O_3_ containing humid air for 24 h (Supplementary Fig. 32). Therefore, PCN-250(Fe_2_Co) represents a rare and excellent catalyst for O_3_ decomposition due to its high stability and activity.

### O_3_ decomposition mechanism

**Fig. 4:**
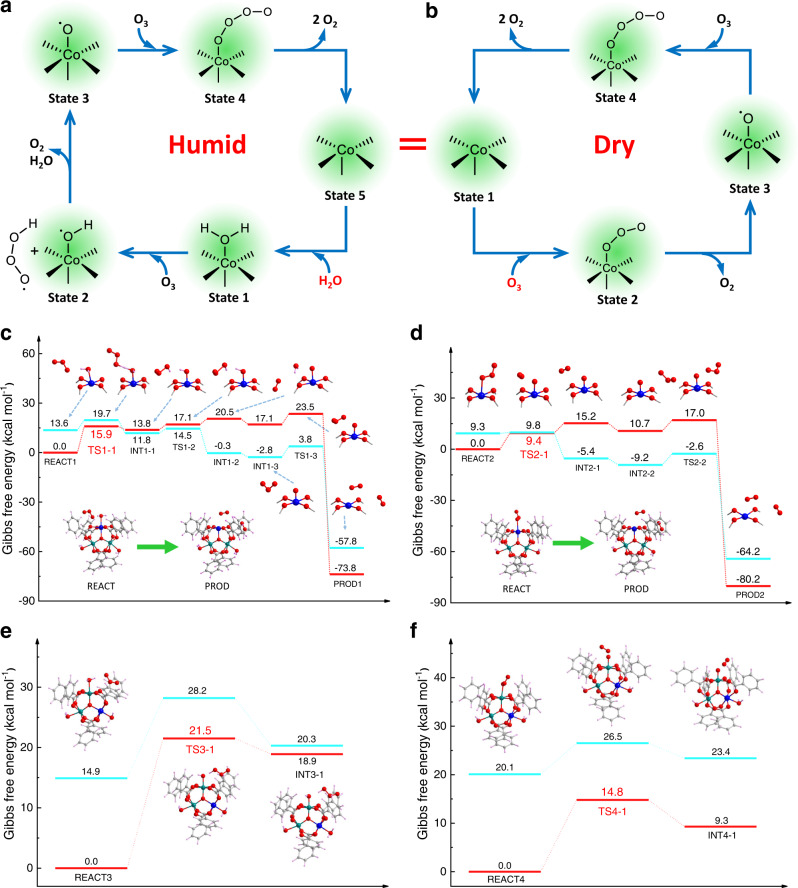
O_3_ decomposition mechanism exploration. Proposed reaction pathways, energy profiles, and stationary-point structures along the calculated reaction pathways for the O_3_ decomposition catalyzed at the Co(II) site of PCN-250(Fe_2_Co) under both **a**, **c** humid and **b**, **d** dry conditions. Energy profiles and stationary-point structures along the calculated reaction pathways at the Fe(III) site of the PCN-250(Fe_2_Co)-catalyzed O_3_ decomposition under **e** humid and **f** dry conditions. Energy levels of the total spin state of S = 14 and S = 16 are shown in red and light blue, respectively. Color code: Co, blue; Fe, turquoise; C, gray; O, red; and H, pink.

To explore the source of the high O_3_ decomposition activity of PCN-250(Fe_2_Co), O_3_ removal tests were also conducted for its starting materials, namely, the H_4_ABTC ligand and the [Fe_2_Co(μ_3_-O)(CH_3_COO)_6_] complex. As shown in Supplementary Fig. 33, the O_3_ removal efficiency of H_4_ABTC was nearly negligible, but the [Fe_2_Co(μ_3_-O)(CH_3_COO)_6_] complex could remove 60–90% O_3_ in inlet gas under dry and humid conditions. These results indicated that the catalytic active sites of PCN-250(Fe_2_Co) for O_3_ decomposition were the metal centers of Fe_2_Co-µ_3_-oxo clusters which are bonding with labile water molecules. It is also revealed that assembling the starting materials into the highly open MOF structure was beneficial to achieve a higher O_3_ removal efficiency due to the increase of accessible catalytic active sites exposed on the pore surface. Since the four PCN-250 MOFs are isomorphous and PCN-250(Fe_2_Co) showed the highest O_3_ removal efficiency, Co(II) center should be the most active catalytic site for O_3_ decomposition among the four types of metal centers (Fe^3+^, Co^2+^, Ni^2+^, and Mn^2+^). In addition, the catalytic reaction mechanisms under humid and dry conditions should be different according to the experimental results. Density functional theory (DFT) calculations were carried out with a [Fe_2_Co(μ_3_-O)(PhCOO)_6_] cluster model to reveal the O_3_ decomposition mechanism at the Co(II) site of PCN-250(Fe_2_Co) and the following reaction pathways were proposed (see supplementary information for details). Under the humid condition, as shown in Fig. 4a, c, the H atom of H_2_O^*^ adsorbed on the Co(II) atom first attacks O_3_ along with the formation of ^•^OOOH and ^*•^OH radicals (^*^ indicates the atom bonded to the Co atom). Subsequently, the remaining H atom of ^*•^OH is transferred to ^•^OOOH generating O_2_, H_2_O, and ^•^O^*^ (State 3 in Fig. 4a and INT1-2, S = 16 in Fig. 4c). Then, another O_3_ molecule attacks ^•^O^*^ releasing two O_2_ molecules. Afterward, the Co(II) atom becomes coordinatively unsaturated and prefers to coordinate with H_2_O over O_3_ under the humid condition due to the favorable adsorption energy of the former (22.4 vs. 9.0 kcal mol^−1^). As a result, the initial state is regenerated, and the catalysis repeats. Throughout the entire catalytic process, the first H transfer is a rate-determining step with a barrier of 15.9 kcal mol^−1^ (TS1-1, S = 14 in Fig. 4c, and Supplementary Table 2).

Differently, under dry conditions, the reaction pathway essentially starts from the coordinatively unsaturated Co(II) center, which is generated by the reaction pathway under humid conditions (from State 1 to State 5 in Fig. 4a). O_3_ first binds to the exposed Co(II) center and then the ^*^O_3_ splits into O_2_ and ^•^O^*^ with a barrier of 9.4 kcal mol^−1^ (State 3 in Fig. 4b, TS2-1, S = 14 in Fig. 4d, and Supplementary Table 3). Afterward, another O_3_ molecule attacks ^•^O^*^, resulting in two O_2_ molecules and a coordinatively unsaturated Co(II) center (State 1 in Fig. 4b, and PROD2, S = 14 in Fig. 4d). The barrier of the rate-determining step in the dry condition is estimated to be 9.4 kcal mol^−1^. It is clear from the above computational results that the rate-determining step in both humid and dry conditions is related to their first elementary reaction, namely the H transfer in the former and the O_3_ decomposition in the latter. Importantly, the barrier of the rate-determining step is smaller in the dry condition than that in the humid condition (9.4 and 15.9 kcal mol^−1^). In addition, it is an endergonic reaction step in the humid condition (∆*G* = 11.8 kcal mol^−1^), while it becomes exergonic in the dry condition (∆*G* = − 5.4 kcal mol^−1^). Taken together, the dry condition is more favorable for the O_3_ removal, which is consistent with the experimental results.

Moreover, we also calculated the rate-determining barriers of the O_3_ decomposition at the Fe(III) site of [Fe_2_Co(μ_3_-O)(PhCOO)_6_] and [Fe_3_(μ_3_-O)(PhCOO)_6_]. In brief, both the Co(II) and Fe(III) sites share a similar catalytic mechanism. However, the Fe(III) site has larger barriers under either humid or dry conditions compared with the Co(II) site. Under humid and dry conditions, the barriers were calculated to be 21.5 and 14.8 kcal mol^−1^ for [Fe_2_Co(μ_3_-O)(PhCOO)_6_] (Fig. 4e, f and Supplementary Table 5); while they were estimated to be 24.7 and 11.7 kcal mol^−1^ for [Fe_3_(μ_3_-O)(PhCOO)_6_] (Supplementary Fig. 34 and Supplementary Table 6). The calculations clearly showed that the enhanced O_3_ decomposition performance of PCN-250(Fe_2_Co) originated from the introduction of open Co(II) sites.

### VOCs adsorption of PCN-250(Fe_2_Co)

**Fig. 5:**
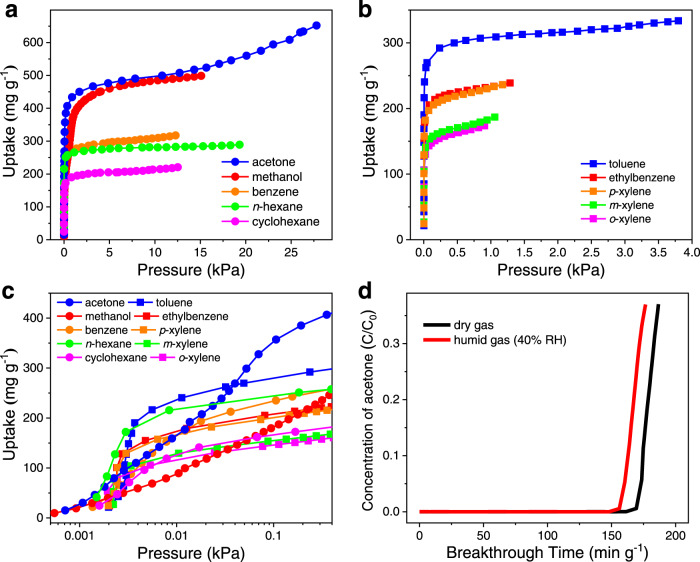
VOCs adsorption studies for PCN-250(Fe_2_Co). **a** for relatively low boiling point VOCs (methanol, acetone, benzene, *n*-hexane, and cyclohexane) and **b** for relatively high boiling point VOCs (toluene, ethylbenzene, *o*-xylene, *m*-xylene, and *p*-xylene) recorded at 25 °C. **c** Zoom-in view for adsorption data at the low-pressure range (below 0.4 kPa). **d** Breakthrough curves for dry and humid air (RH = 40% at room temperature) containing acetone vapor (C_0_ = 3000 ppm) flowed through a column packed with PCN-250(Fe_2_Co). Test conditions: 0.02 g PCN-250(Fe_2_Co) diluted with 0.4 g quartz sand, concentration of acetone = 3000 ppm, flow rate = 0.2 L min^−1^, room temperature.

Due to its high porosity and moderate pore size, PCN-250(Fe_2_Co) is potentially capable of removing other pollutants in the air by adsorption besides O_3_. VOCs are not only a class of hazardous air pollutants but also the precursor of O_3_, the removal of VOCs from air is thus of high importance^35^. To evaluate its performance on VOCs removal, adsorption isotherms of PCN-250(Fe_2_Co) were recorded at 25 °C for ten common VOCs, including methanol, acetone, two aliphatic hydrocarbons (*n*-hexane and cyclohexane) and six aromatic hydrocarbons (benzene, toluene, ethylbenzene, *o*-xylene, *m*-xylene, and *p*-xylene). Type-I adsorption isotherms were obtained for all the tested VOCs (Fig. 5a, b), showing an abrupt increase of uptakes at low vapor pressures below *P/P*_0_ = 0.002 (Supplementary Fig. 35). At 0.01 kPa, the uptakes of methanol, acetone, *n*-hexane, cyclohexane, benzene, toluene, ethylbenzene, *o*-xylene, *m*-xylene, and *p*-xylene were 89, 177, 216, 123, 175, 241, 179, 114, 130, and 164 mg g^−1^ (Fig. 5c), respectively, corresponding to 18–75% of the maximum uptakes at 0.90‒0.99 *P/P*_0_ (174‒652 mg g^−1^). The high VOCs adsorption capacities of PCN-250(Fe_2_Co) suggest that the MOF may serve as a general adsorbent to remove various VOCs even if the concentrations of VOCs are low. In practical application, the humidity of air can significantly affect the performance of adsorbents for VOCs removal. To check the effect of humidity on PCN-250(Fe_2_Co), breakthrough experiments of dry and humid air flows containing 3000 ppm acetone vapor passing through a column of the MOF adsorbent (20 mg) were carried out at room temperature (see supplementary information for details). As shown in Fig. 5d, for dry gas, acetone started to penetrate the MOF column after about 190 s (160 min g^−1^), corresponding to an acetone capture capacity of ca. 355 mg g^−1^ in the dynamic adsorption process, which is close to the theoretical uptake at 0.3 kPa (400 mg g^−1^) from the static adsorption isotherm measurement. When the humidity of the gas mixture increased to RH = 40%, about 93% of the acetone capture capacity of PCN-250(Fe_2_Co) retained, suggesting a good tolerance to the humidity of the MOF adsorbent for acetone vapor capture. Moreover, PXRD measurements revealed that PCN-250(Fe_2_Co) also showed excellent stability toward the VOCs (Supplementary Fig. 36).

It is noteworthy that previously reported materials showing high performance on catalytic O_3_ removal, such as the above-mentioned ZZU-281 and some advanced transition metal oxides^19,22,24^, are of low porosity or nonporous and thus have limited capacity to adsorptively remove non-O_3_ air pollutants like VOCs. In addition, compared with the most common and prevalent adsorbent for VOCs removal, activated charcoal, PCN-250(Fe_2_Co), shows more stable performance and higher activity on catalytic O_3_ removal. The high O_3_ removal performance, high porosity, high stability, and facile preparation make PCN-250(Fe_2_Co) a unique and promising multifunctional material for air purification.

## Discussion

The great potential of isomorphous bimetallic MOFs PCN-250(Fe_2_M) as high-performance porous catalysts for both O_3_ degradation and VOCs removal is demonstrated. Particularly, PCN-250(Fe_2_Co) showed 100% O_3_ removal efficiency for a continuous airflow containing 1 ppm O_3_ over a wide humidity range (0‒80% RH) at room temperature. Under the same conditions, the O_3_ removal efficiency of PCN-250(Fe_2_Co) was higher than those of many representative materials, including *α*-Fe_2_O_3_, Co_3_O_4_, CoFe_2_O_4_, activated charcoal, and MIL-100(Fe). PCN-250(Fe_2_Co) also showed higher catalytic activity for O_3_ decomposition than ZZU-281 under dry conditions. The highly porous and crystalline structure of PCN-250(Fe_2_Co) remained intact after it was treated by soaking in water for 30 days, or exposing to ambient air for 4 months at room temperature, and its O_3_ decomposition capacity retained after exposure of the MOF to relatively high concentration O_3_ in dry or humid air (50 ppm). The O_3_ decomposition reaction mechanisms catalyzed by PCN-250(Fe_2_Co) under dry and wet conditions were proposed based on DFT calculations. It was suggested that the high catalytic performance of PCN-250(Fe_2_Co) originated from the introduction of open Co(II) sites as well as its porous structure. Meanwhile, PCN-250(Fe_2_Co) is highly potential in the adsorptive removal of VOCs from the air, which is not only a class of hazardous air pollutants but also the precursor of O_3_. It is an attribution not available for the other O_3_-decomposition catalysts. At a low vapor pressure round 10 Pa and room temperature, PCN-250(Fe_2_Co) showed high adsorption capacities (89‒241 mg g^−1^) for ten common VOCs, namely methanol, acetone, *n*-hexane, cyclohexane, benzene, toluene, ethylbenzene, *o*-xylene, *m*-xylene, and *p*-xylene. The VOC capture capacity of PCN-250(Fe_2_Co) from both dry and humid air was confirmed by breakthrough experiments. PCN-250(Fe_2_Co) may serve as a unique and promising multifunctional material for air purification. This work opens up a new avenue to develop new materials for air pollution control, and the introduction of high porosity to O_3_-decomposition catalysts may endow the O_3_-decomposition catalysts with multifunctionality, like adsorption, separation, and sensing, which could be beneficial in practical air purification applications.

## Methods

### Synthesis of PCN-250(Fe_2_M) (M = Fe, Co, Ni, Mn)

PCN-250(Fe_2_M) was synthesized following the literature with some modifications^40^. Trinuclear complexes [Fe_2_M(μ_3_-O)(CH_3_COO)_6_] (M = Fe, Co, Ni, Mn) (45 mg), H_4_ABTC (30 mg), and acetic acid (2.0 mL) were ultrasonically dissolved in DMF (6 mL) in a 15 mL high-pressure vessel. The vessel was then tightly sealed, stirred, and heated in an oil bath of 140 °C for 12 h. After cooling down to room temperature, the as-synthesized solid was washed successively with DMF (3 × 40 mL) at 80 °C for 48 h and methanol (3 × 40 mL) at 60 °C for 48 h, and then dried under vacuum at 80 °C for 8 h.

### O_3_ decomposition measurement

Catalytic O_3_ decomposition experiments were conducted with continuous-flow fixed-bed reactors (quartz tube, inner diameter: 4 mm; length: 15 cm) of the tested materials at room temperature (unless otherwise specified) and the inlet gas flow rate of 1 L min^−1^. Before the test, all tested samples were dried in a vacuum at 80 °C for 12 h. Then a mixture of 30 mg tested materials and 200 mg quartz sand (40–60 mesh) were loaded into the fixed-bed reactors and immobilized by quartz wool. A humidity generator was used to control the humidity of inlet gas. The temperature of the reactor was maintained by a thermostatic water bath. O_3_ in the inlet gas was generated by passing zero air through an ultraviolet lamp, and its concentration was controlled by the flow rate of zero air. The O_3_ concentration in inlet and outlet gas was detected by an O_3_ monitor with a detection limit of 2 ppb. O_3_ removal efficiency was calculated as 100% × (C_inlet_ – C_outlet_)/C_inlet_. A schematic representation of the O_3_ decomposition measurement setup is shown in Supplementary Fig. 14.

### Breakthrough experiments

The breakthrough experiments were carried out with zero air containing ca. 3000 ppm acetone vapor by a setup shown in Supplementary Fig. 15. Quartz tubes (4 mm inner diameter and 15 cm length) were packed with a mixture of 20 mg PCN-250(Fe_2_Co) and 400 mg quartz sand (40–60 mesh). A humidity generator was used to control the humidity of inlet gas (flow rate = 198 mL min^−1^). The flow rate of air containing saturated acetone vapor at room temperature was controlled by a mass flow controller (Alicat, flow rate = 2 mL min^−1^). The concentration of acetone in the gases passing through the MOF column was monitored with a Hiden HPR20 mass spectrometer gas analysis system.

### Theoretical calculation

A PCN-250(Fe_2_Co) cluster model is constructed by cutting from its periodic structure as done in recent works^30^ (Supplementary Fig. 2a), and it includes the Fe_2_Co core, six phenyl carboxylic groups coordinated with one Co atom and two Fe atoms, and two water molecules occupying two exposed Fe sites. All calculations presented in this work are performed using the unrestricted density functional theory (DFT) with the hybrid functional PBE0 as implemented in the Gaussian09 package^57–59^. Geometry optimizations are carried out with the LANL2DZ basis sets for the Fe and Co atoms and the 6-31 G* basis sets for the other atoms^60–62^. Dispersion correction is included in geometry optimizations using the empirical formula by Grimme et al.^63^. Frequency calculations are performed at the same level of theory to confirm the nature of stationary points and to obtain zero-point energies (ZPE) and entropy effects. The reported free energies are corrected for dispersion, ZPE, and entropy effects.

## Supplementary information

Supplementary Information

## Data Availability

All data supporting the findings of this study are available within the article and its Supplementary Information, or from the corresponding author upon reasonable request.
