# Supplementary material for: Catalytic ozone decomposition and adsorptive VOCs removal in bimetallic metal-organic frameworks
Source: Nat Commun. 2022 Aug 25;13:4991. doi: 10.1038/s41467-022-32678-2 (PMC9411195; doi:10.1038/s41467-022-32678-2)
Supplement: Supplementary file 1 — Supplementary Information [file 41467_2022_32678_MOESM1_ESM.pdf]

Supplementary Information

Catalytic Ozone Decomposition and Adsorptive VOCs Removal in Bimetallic Metal-Organic Frameworks

Chen Dong[1‡], Jia-Jia Yang[2‡], Lin-Hua Xie[1*], Ganglong Cui[2*], Wei-Hai Fang[2], Jian-Rong Li[1*]

[1]Beijing Key Laboratory for Green Catalysis and Separation, and Department of Environmental Chemical Engineering, Beijing University of Technology, Beijing, 100124, China

[2]Key Laboratory of Theoretical and Computational Photochemistry, Ministry of Education, College of Chemistry, Beijing Normal University, Beijing 100875, China.

[‡]These authors contributed equally: Chen Dong, Jia-Jia Yang.

[*]Email: xielinhua@bjut.edu.cn; ganglong.cui@bnu.edu.cn; jrli@bjut.edu.cn

Supplementary Methods

Chemicals and instruments

Sodium hydroxide, 5-nitroisophthalic acid, glucose, hydrochloric acid, sodium acetate trihydrate, iron(III) nitrate, manganese(II) nitrate, cobalt(II) nitrate, nickel(II) nitrate, dimethylformamide, acetic acid, and methanol were purchased from Sinopharm Chemical Reagent Co. Ltd. or Beijing Chemical Reagent Company. Activated charcoal (Sigma-Aldrich Supelco), Trimesic Acid Trimethyl Ester (Aladdin), $CoFe_2O_4$ (Macklin), α-Fe_2O_3(Macklin), and Co_3O_4 (Macklin) were used as purchased without further purification. 8-watt UV lamp was bought from Beijing Aerospace HONGDA Optoelectronics Technology Co. Ltd. Ozone monitor (Model 205, limit of detection: 2 ppb; resolution: 0.1 ppb) was bought from 2B Technologies. Humidity generator (HSDG-A) was bought from Suzhou Huaxiangshida Environmental Protection Technology Co., Ltd. Ozone test strips (Model 907 36) were bought from MACHEREY-NAGEL GmbH & Co.KG.

The powder X-ray diffraction (PXRD) patterns were recorded on a Rigaku Smartlab3 X-ray Powder Diffractometer equipped with a Cu-sealed tube (λ = 1.54178 Å). N_2 adsorption/desorption isotherms were measured by using a Micrometritics BELSORP MAX II Surface Characterization Analyzer at 77 K. ^{1}H NMR data were recorded on a Bruker Avance 400 MHz spectrometer. An IR Affinity-1 instrument was used for recording Fourier transform infrared (FT-IR) spectra. Thermogravimetric analysis (TGA) data were obtained on a TGA-50 (Shimadzu) thermogravimetric analyzer with heating from 25 to 700 °C (10 °C min^{-1}) under air atmosphere. X-ray photoelectron spectroscopy (XPS) measurements were carried out using an ESCALAB 250 instrument. Metal contents of MOFs were determined by ICP-AES on an atomic emission spectrometer (OPTIMA7000DV, PE, AGILENT, America). The water contact angles were measured on the contact angle system JY-82C (Chengde Dingsheng, China).

Synthesis of MOFs

Synthesis of MIL-100(Fe): $FeCl_3 \cdot 6H_2O$ (162.2 mg), 1,3,5-benzenetricarboxylate (138.7 mg) and deionized water (5 mL) were put into a 20 mL Teflon-lined autoclave and heated at 130 °C for 72 h.[1] After cooling down to room temperature, the as-synthesized solid was washed with ethanol (3 × 40 mL) at 60 °C for 48 h and dried under vacuum at 80 °C for 8 h.

Synthesis of ZZU-281: $Mn(NO_3)_2 \cdot 6H_2O$ (114.8 mg), H_4TTPE ligand (55.6 mg) were ultrasonically dissolved in the mixture of DMA (4 ml) and deionized water (2 mL) in a 20 mL Teflon-lined autoclave and heated at 130 °C for 48 h.[2] After cooling down to room temperature, the as-synthesized solid was washed successively with DMF (3 × 40 mL) at 80 °C for 48 h and methanol (3 × 40 mL) at 60 °C for 48 h, and then dried under vacuum at 80 °C for 8 h.

Synthesis of MIL-101(Cr): $Cr(NO_3)_3 \cdot 9H_2O$ (400 mg), terephthalic acid (166 mg), deionized water (4.75 mL), and HF (20 μL, 48-51 wt%) were put into a 20 mL Teflon-lined autoclave and heated at 200 °C for 8 h.[3] After cooling down to room temperature, the as-synthesized solid was washed successively with 100 mL 95:5 EtOH:H_2O (v/v) solution at 80 °C for 24 h, NH_4F solution (90 mL, 30 mmol L^{-1}) at 70 °C for 24 h, and deionized water (70 mL) at 90 °C for 3h. The resulting solid was further washed with acetone for three times, and then dried under vacuum at 80 °C for 12 h.

Synthesis of ZIF-8: $Zn(NO_3)_2 \cdot 6H_2O$ (1.17 g) and 2-methylimidazole (22.70 g) were dissolved in 8 and 80 mL deionized water respectively, then the aqueous solution of zinc nitrate was added into the aqueous solution of 2-methylimidazole under stirring at room temperature.[4] After stirring for 5 min, the product was collected by centrifuging, washed with deionized water (3 × 40 mL) for 48 h and methanol (3 × 40 mL) for 48 h, and then dried under vacuum at 80 °C for 8 h.

Synthesis of ZIF-L: $Zn(NO_3)_2 \cdot 6H_2O$ (0.59 g) and 2-methylimidazole (1.30 g) were dissolved in deionized water (40 mL) respectively, then the aqueous solution of zinc

nitrate was added into the aqueous solution of 2-methylimidazole under stirring at room temperature.[5] After stirring for 4 h, the product was collected by centrifuging, washed with deionized water (3 × 40 mL) for 48 h and methanol (3 × 40 mL) for 48 h, and then dried under vacuum at 80 °C for 8 h.

Computational details

It is commonly known that there are several different oxidation states available for both Fe and Co atoms, which can lead to distinct spin multiplicities for the PCN-250(Fe_2Co) model (Supplementary Fig. 1). Thus, we first need to decide the most stable spin state, from which the catalytic reaction starts to proceed. A series of DFT calculations show that it has the lowest energy when the total spin multiplicity is 14 (i.e. 13 unpaired electrons, see Supplementary Table 1). Electronic structure analysis shows that oxidation states of the Fe and Co atoms are +3 and +2 in the cluster model of PCN-250(Fe_2Co), respectively. This means that 10 unpaired electrons singly occupy 10 3d orbitals of the two Fe(III) atoms and 3 unpaired electrons singly occupy 3 3d orbitals of the Co(II) atom. In such situation, there are two doubly occupied 3d orbitals of the Co(II) atom. Interestingly, all unpaired electrons are mainly located on metal atoms and do not involve ligands, which is supported by the calculated spin density plotted in Supplementary Fig. 1b.

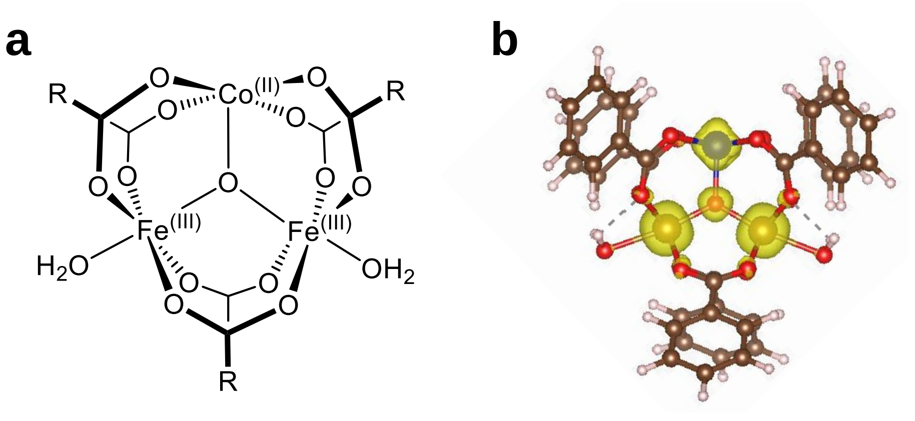

Supplementary Fig. 1. The cluster model of PCN-250(Fe_2Co). a The cluster model (R = phenyl groups) used to study the catalytic mechanism of O_3 decomposition under dry and humid conditions. **b** the calculated spin density plot with a total spin multiplicity of 14.

Starting from the ground spin state of S = 14, we have separately calculated corresponding catalytic reaction paths of O_3 decomposition. First, we focus on the reaction mechanism under humid condition. In such case, a water molecule first occupies the Co(II) atom, this process is favorable thermodynamically with an adsorption energy of 22.4 kcal mol^{-1}. Moreover, the water adsorption does not change the lowest spin state (still S = 14). As shown in Fig. 4a and 4c in the maintext, starting this H_2O-coordinated PCN-250(Fe_2Co) complex, the first step is a hydrogen transfer reaction process from H_2O^* to O_3, i.e. from REACT1 to INT1-1, which produces $^•$OOOH and $^{*•}$OH (* stands for the atom that is coordinated with the Co atom). It requires 15.9 kcal mol^{-1} energy to overcome the energy barrier at TS1-1 in the lowest spin state with a total spin multiplicity of 14 (S = 14). Although this process becomes easier in the spin state with S = 16, this path is unimportant because of much higher energy of reactant. Interestingly, there is a crossing point between the two spin states (S = 14 and 16) in the vicinity of INT1-1 and their energies are calculated to be 11.8 and 13.8 kcal mol^{-1}, respectively. Thus, near INT1-1, the system will hop to the spin state with S = 16 via an intersystem crossing process. Subsequently, further hydrogen transfer from $^{*•}$OH to $^•$OOOH to produce O_2, H_2O, and $^•O^*$ takes place by overcoming a barrier of 2.5 kcal mol^{-1}. Here, the reason for the final formation of $^•O^*$ is that an electron of Co is bonded with an electron of $^•O^*$. The bond order is 1.16 and the bond length is 1.62 Å. Finally, a new O_3 molecule attacks the $^•O^*$ atom coordinated with the Co atom generating two triplet O_2 molecules, which demands 6.6 kcal mol^{-1} energy to overcome the barrier at TS1-3 leading to a complex of PCN-250(Fe_2Co) and two triplet O_2 molecules. Once these two O_2 molecules leave away, the original PCN-250(Fe_2Co) catalyst is recovered with the lowest spin state of S = 14. Therefore, this step must involve a spin flip process after the system overcomes the barrier at TS1-3. To sum up, the rate-determining step is the first hydrogen transfer reaction in the spin state of S = 14 with a barrier of 15.9 kcal mol^{-1}; the entire catalytic reaction is allowed thermodynamically because of releasing 73.8 kcal mol^{-1} energy; importantly, the reaction involves two spin states and takes place in a nonadiabatic means.

Second, under dry condition, O_3 is directly adsorbed on the exposed Co(II) atom with an adsorption energy of 9.0 kcal mol^{-1}. Note that the spin state of S = 14 is still the lowest upon O_3 adsorption. As shown in Fig. 4b and 4d in the maintext, the first step involves the O-O bond fission of $^{*}O_3$ coordinated with the Co atom producing one triplet O_2 molecule and $^{\bullet}O^{*}$; thus, it must encounter a spin-flip process. The corresponding spin crossing point is determined and its energy is 9.4 and 9.8 kcal mol^{-1} in the spin states of S = 14 and S = 16, respectively. Near this crossing point, the system decays to the spin state of S = 16 and the O-O bond is simultaneously dissociated to produce one O_2 molecule and $^{\bullet}O^{*}$ coordinated with the Co atom. Subsequently, as with the above situation under humid condition, a new O_3 molecule attacks the $^{\bullet}O^{*}$ atom generating a complex of PCN-250(Fe_2Co) and two triplet O_2 molecules, which demands 6.6 kcal mol^{-1} energy to overcome the barrier at TS2-2. The leaving of two triplet O_2 molecules recovers PCN-250(Fe_2Co) with the lowest spin state of S = 14. To summarize, the O-O bond cleavage is the rate-determining step throughout the entire reaction under dry condition (9.4 kcal mol^{-1}); similarly, the entire catalytic reaction releases 80.2 kcal mol^{-1} energy, involves two spin states, and takes place nonadiabatically.

Under humid condition, all three metal centers are coordinated with water at initial state (Fig. 4c in the maintext), but under dry condition, only Co center is uncoordinated (Fig. 4d in the maintext). The construction of such an uncoordinated model was based on the following considerations. Under dry condition, the Co(II) and Fe(III) centers are all coordinated with water at the very beginning. The O_3 decomposition reaction can happen on either the H_2O-coordinated Co(II) sites or the H_2O-coordinated Fe(III) sites. In the former case, the reaction proceeds following the pathway shown in Fig. 4a in the maintext, producing a coordinatively unsaturated Co(II) site (State 5). The energy barrier of the rate-determining step is calculated to be 15.9 kcal mol^{-1} (Fig. 4c in the maintext). In the latter case, the O_3 decomposition reaction proceeds in a similar way, but the energy barrier of the rate-determining step (21.5 kcal mol^{-1}, Fig. 4e in the maintext) is higher than that in the former case. Therefore, the formation of a cluster

with one coordinatively unsaturated Co(II) site and two H_2O-coordinated Fe(III) sites is energetically favorable. After the coordinatively unsaturated Co(II) site is formed, the O_3 decomposition reaction would happen on the open Co(II) site following the reaction pathway shown in Fig. 4b in the maintext, because the energy barrier of the rate-determining step becomes much lower (9.4 kcal mol^{-1}, Fig. 4d in the maintext). In short, under dry condition the starting model is set to contain one open Co(II) site and two H_2O-coordinated Fe(III) sites by considering that the formation of such a model is more energetically favorable than the formation of other possible models.

Supplementary Tables and Figures

Supplementary Table 1. Energies of PCN-250(Fe_2Co) in various electronic states.

Multiplicity	PCN-250(Fe_2Co) Energy (kcal mol^{-1})
**2**	59.1
**4**	41.0
**6**	33.67
**8**	36.12
**10**	24.6
**12**	17.89
**14**	0
**16**	79.6
**18**	159.2

Supplementary Table 2. Energies of key structures along the catalytic path of Co(II) in PCN-250(Fe_2Co) under humid condition in different spin states (S = 14, 16, and 18).

Energy (kcal mol^{-1})	14	16	18	20
**REACT1**	0	13.6	89.4	
**TS1-1**	15.9	19.7		
**INT1-1**	13.8	11.8	87.0	
**TS1-2**	17.1	14.5		
**INT1-2**	20.5	-0.3	4.2	
**INT1-3**	17.1	-2.8	2.9	
**TS1-3**	23.5	3.8		
**PROD1**	-73.8	-57.8	-55.8	-90.0

Supplementary Table 3. Energies of key structures along the catalytic path of Co(II) in PCN-250(Fe_2Co) under dry condition in different spin states (S = 14, 16, and 18).

**Energy (kcal mol^{-1})**	**14**	**16**	**18**	**20**
**REACT2**	0	9.3	81.5	
**TS2-1**	9.4	9.8		
**INT2-1**	15.2	-5.4	-0.9	
**INT2-2**	10.7	-9.2	9.4	
**TS2-2**	17.0	-2.6		
**PROD2**	-80.2	-64.2	-62.3	-96.5

Supplementary Table 4. Energies of PCN-250(Fe_3) in various electronic states.

Multiplicity	PCN-250(Fe_3) Energy (kcal mol^{-1})
**2**	67.4
**4**	69.4
**6**	53.0
**8**	45.5
**10**	54.3
**12**	33.5
**14**	15.2
**16**	0

Supplementary Table 5. Energies of key structures along the catalytic path of Fe(III) in PCN-250(Fe_2Co) under humid and dry conditions in different spin states (S = 14, 16 and 18).

humid condition			dry condition			
**Energy (kcal mol^{-1})**	**14**	**16**	**Energy (kcal mol^{-1})**	**14**	**16**	**18**
**RACT3**	0	14.9	**RACT4**	0	20.1	95.0
**TS3-1**	21.5	28.2	**TS4-1**	14.8	26.5	
**INT3-1**	18.9	20.3	**INT4-1**	9.3	23.4	6.8

Supplementary Table 6. Energies of key structures along the catalytic path of Fe(III) in PCN-250(Fe_3) under humid and dry conditions in different spin states (S = 16, 18 and 20).

humid condition			dry condition			
**Energy (kcal mol^{-1})**	**16**	**18**	**Energy (kcal mol^{-1})**	**16**	**18**	**20**
**RACT5**	0	18.1	**RACT6**	0	18.7	93.6
**TS5-1**	24.7	26.4	**TS6-1**	11.7	19.1	98.5
**INT5-1**	27.5	18.6	**INT6-1**	16.5	22.2	8.2

Supplementary Table 7. ICP-AES analysis results for PCN-250 samples.

Sample	Iron (Fe) percentage	Metal (M) percentage	Ratio of Fe to M
**PCN-250(Fe_3)**	100		
**PCN-250(Fe_2Co)**	66	34	1.94
**PCN-250(Fe_2Ni)**	67	33	2.03
**PCN-250(Fe_2Mn)**	68	32	2.13

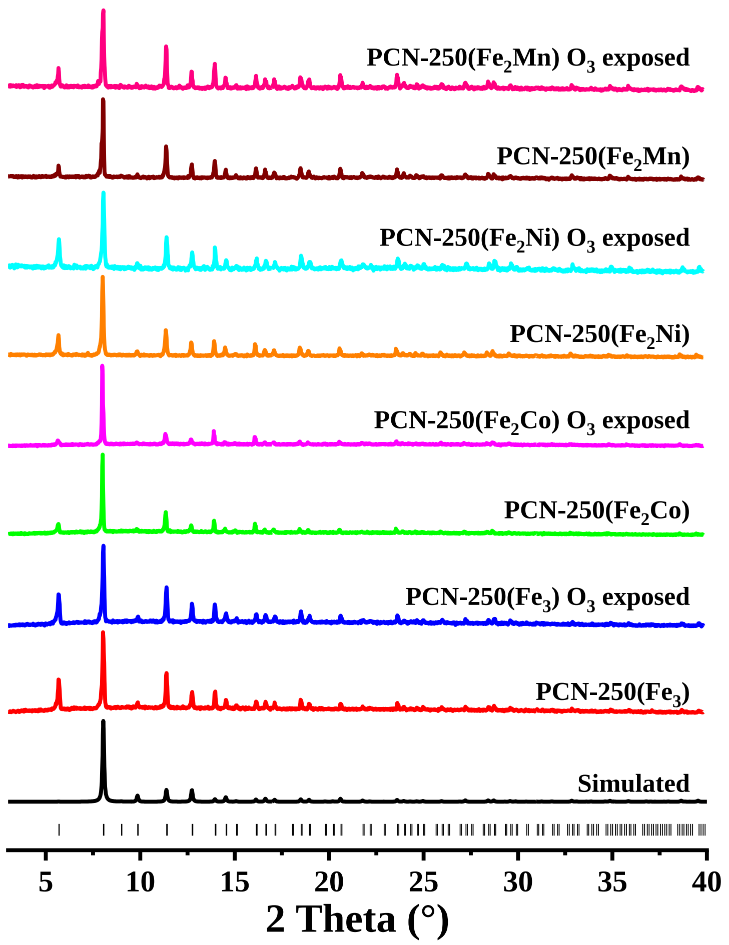

Supplementary Fig. 2. PXRD patterns of PCN-250. PXRD patterns of simulated, pristine and O_3 exposed PCN-250 samples. The MOF samples were exposed to a continuous O_3-containing humid air flow (RH = 40%; flow rate = 0.5 L min^{-1}; concentration of O_3 = 50 ppm) for 50 hours, and then to a continuous O_3-containing dry air flow (RH < 1%; flow rate = 0.5 L min^{-1}; concentration of O_3 = 50 ppm) for another 50 hours at room temperature.

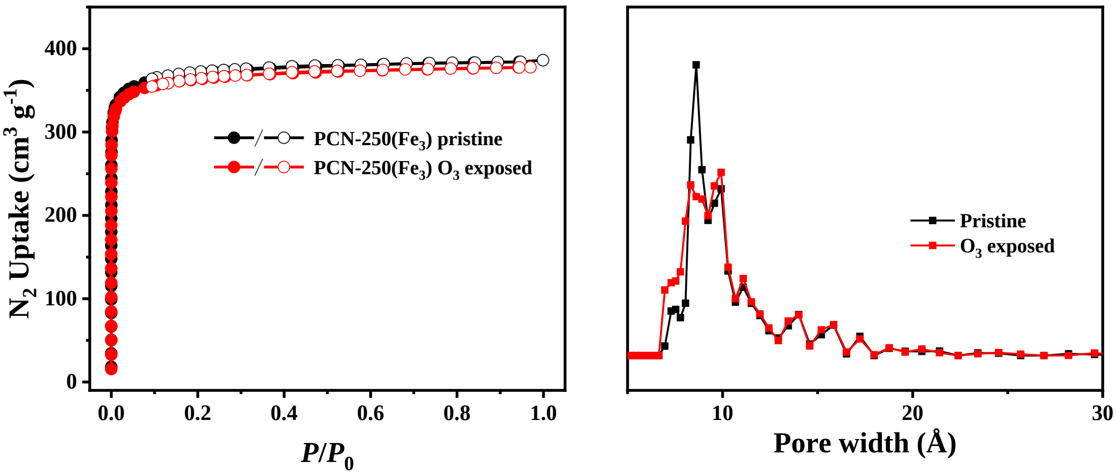

Supplementary Fig. 3. Porosity of PCN-250(Fe$_3$). N_2 adsorption isotherms and pore size distributions of pristine and O_3 exposed PCN-250(Fe$_3$) samples recorded at 77 K. The O_3 exposed MOF sample was treated by a continuous O_3-containing humid air flow (RH = 40%; flow rate = 0.5 L min^{-1}; concentration of O_3 = 50 ppm) for 50 hours, and then by a continuous O_3-containing dry air flow (RH < 1%; flow rate = 0.5 L min^{-1}; concentration of O_3 = 50 ppm) for another 50 hours at room temperature.

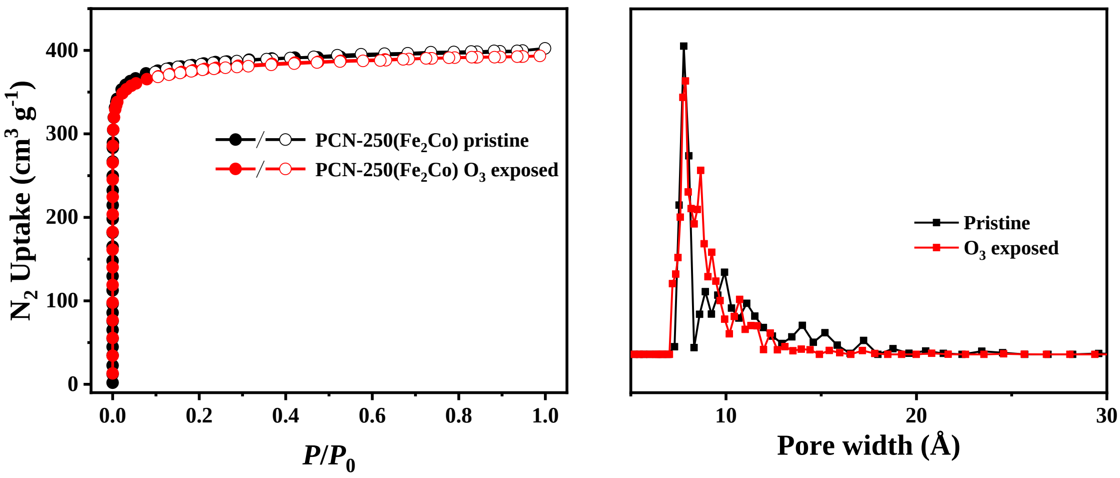

Supplementary Fig. 4. Porosity of PCN-250(Fe_2Co). N_2 adsorption isotherms and pore size distributions of pristine and O_3 exposed PCN-250(Fe_2Co) samples recorded at 77 K. The O_3 exposed MOF sample was treated by a continuous O_3-containing humid air flow (RH = 40%; flow rate = 0.5 L min^{-1}; concentration of O_3 = 50 ppm) for 50 hours, and then by a continuous O_3-containing dry air flow (RH < 1%; flow rate = 0.5 L min^{-1}; concentration of O_3 = 50 ppm) for another 50 hours at room temperature.

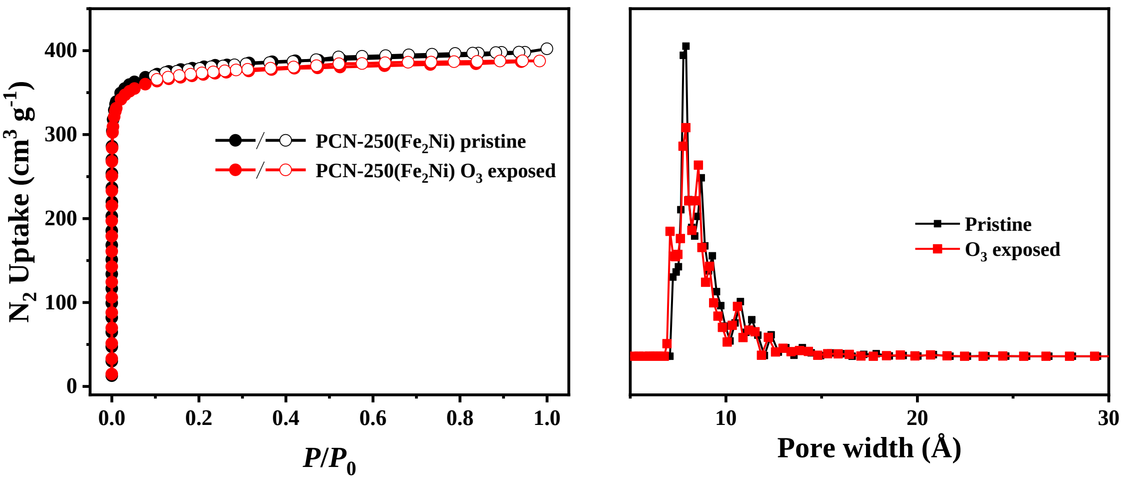

Supplementary Fig. 5. Porosity of PCN-250(Fe$_2$Ni). N_2 adsorption isotherms and pore size distributions of pristine and O_3 exposed PCN-250(Fe$_2$Ni) samples recorded at 77 K. The O_3 exposed MOF sample was treated by a continuous O_3-containing humid air flow (RH = 40%; flow rate = 0.5 L min^{-1}; concentration of O_3 = 50 ppm) for 50 hours, and then by a continuous O_3-containing dry air flow (RH < 1%; flow rate = 0.5 L min^{-1}; concentration of O_3 = 50 ppm) for another 50 hours at room temperature.

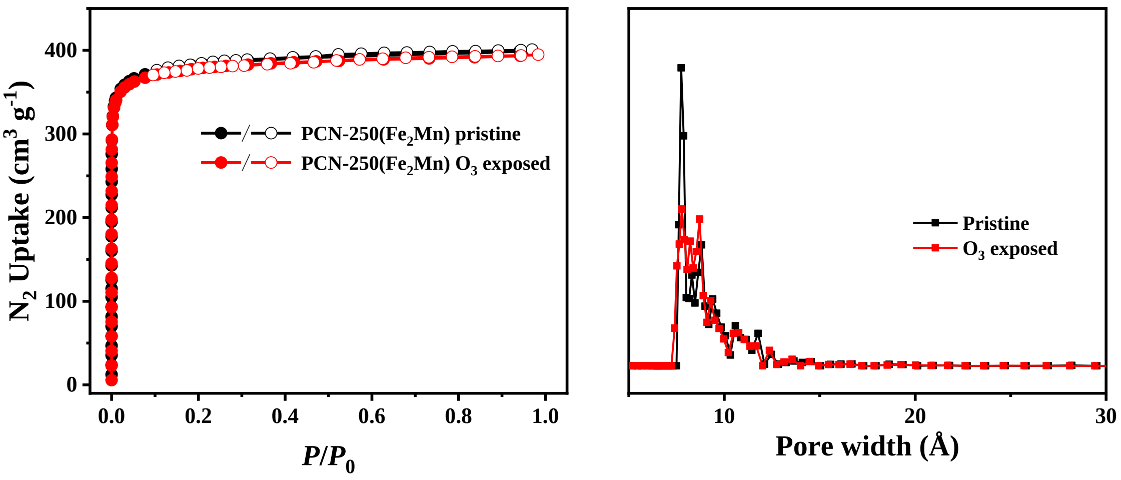

Supplementary Fig. 6. Porosity of PCN-250(Fe$_2$Mn). N_2 adsorption isotherms and pore size distributions of pristine and O_3 exposed PCN-250(Fe$_2$Mn) samples recorded at 77 K. The O_3 exposed MOF sample was treated by a continuous O_3-containing humid air flow (RH = 40%; flow rate = 0.5 L min^{-1}; concentration of O_3 = 50 ppm) for 50 hours, and then by a continuous O_3-containing dry air flow (RH < 1%; flow rate = 0.5 L min^{-1}; concentration of O_3 = 50 ppm) for another 50 hours at room temperature.

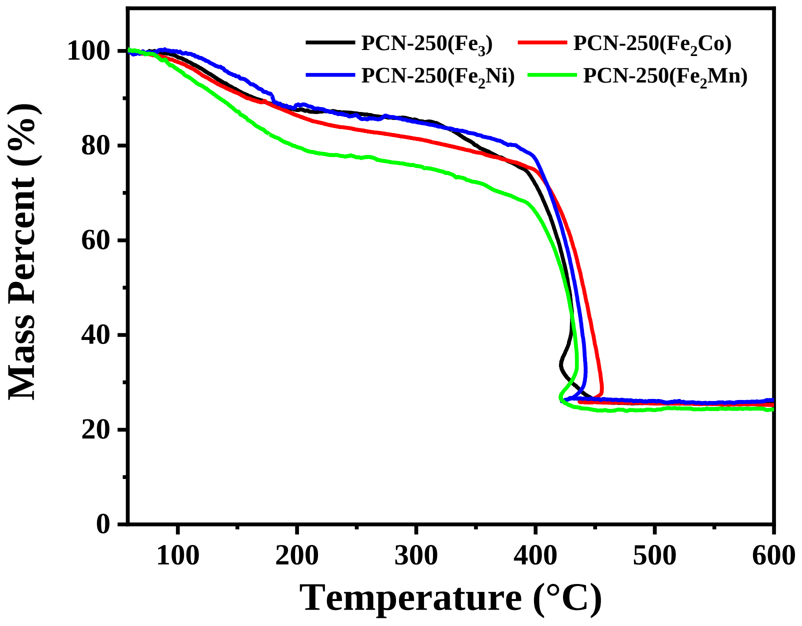

Supplementary Fig. 7. TGA curves. The TGA curves of PCN-250 samples recorded under air flow.

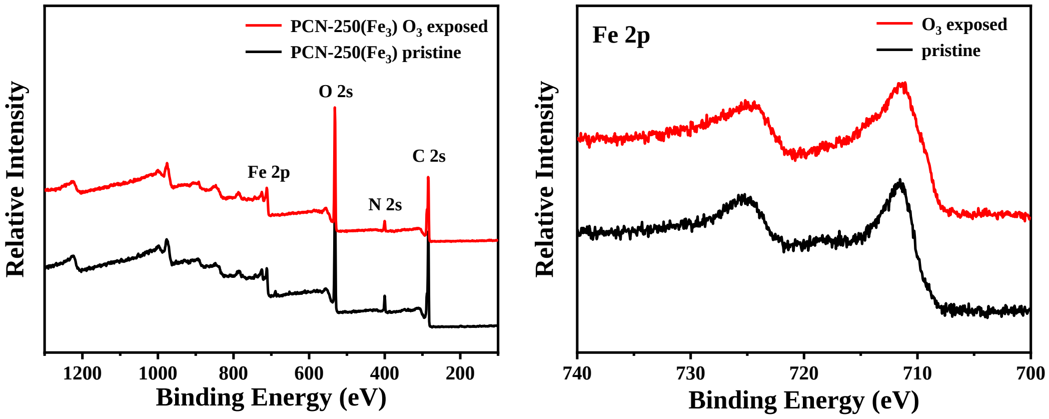

Supplementary Fig. 8. XPS spectra for PCN-250(Fe_3). The XPS spectra of pristine and O_3 exposed PCN-250(Fe_3). The MOF sample was exposed to a continuous O_3-containing humid air flow (RH = 40%; flow rate = 0.5 L min^{-1}; concentration of O_3 = 50 ppm) for 50 hours, and then to a continuous O_3-containing dry air flow (RH < 1%; flow rate = 0.5 L min^{-1}; concentration of O_3 = 50 ppm) for another 50 hours at room temperature.

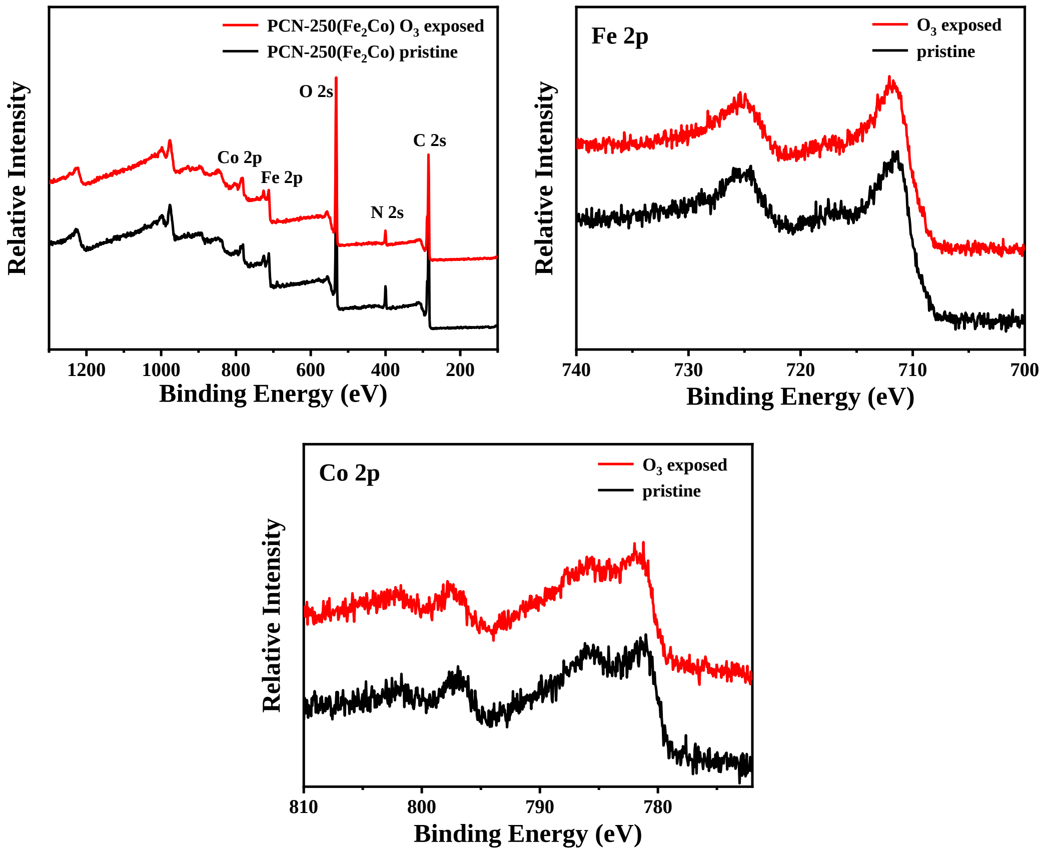

Supplementary Fig. 9. XPS spectra for PCN-250(Fe_2Co). The XPS spectra of pristine and O_3 exposed PCN-250(Fe_2Co). The MOF sample was exposed to a continuous O_3-containing humid air flow (RH = 40%; flow rate = 0.5 L min^{-1}; concentration of O_3 = 50 ppm) for 50 hours, and then to a continuous O_3-containing dry air flow (RH < 1%; flow rate = 0.5 L min^{-1}; concentration of O_3 = 50 ppm) for another 50 hours at room temperature.

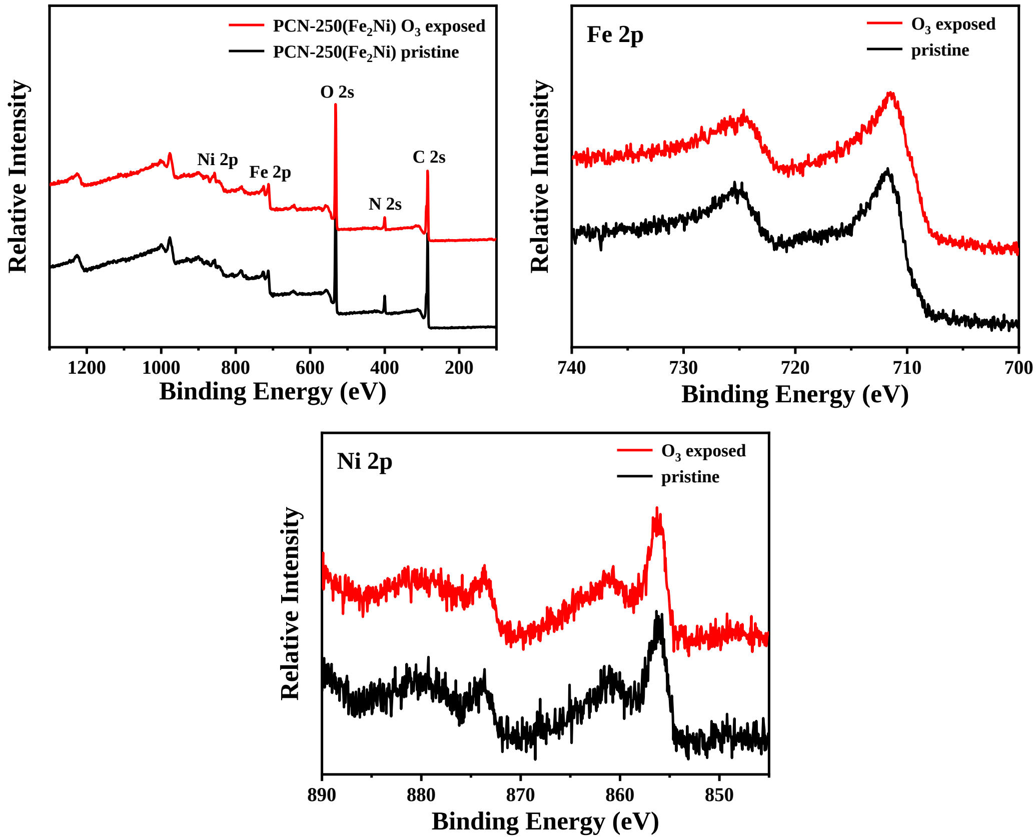

Supplementary Fig. 10. XPS spectra for PCN-250(Fe2Ni). The XPS spectra of pristine and O_3 exposed PCN-250(Fe2Ni). The MOF sample was exposed to a continuous O_3-containing humid air flow (RH = 40%; flow rate = 0.5 L min^{-1}; concentration of O_3 = 50 ppm) for 50 hours, and then to a continuous O_3-containing dry air flow (RH < 1%; flow rate = 0.5 L min^{-1}; concentration of O_3 = 50 ppm) for another 50 hours at room temperature.

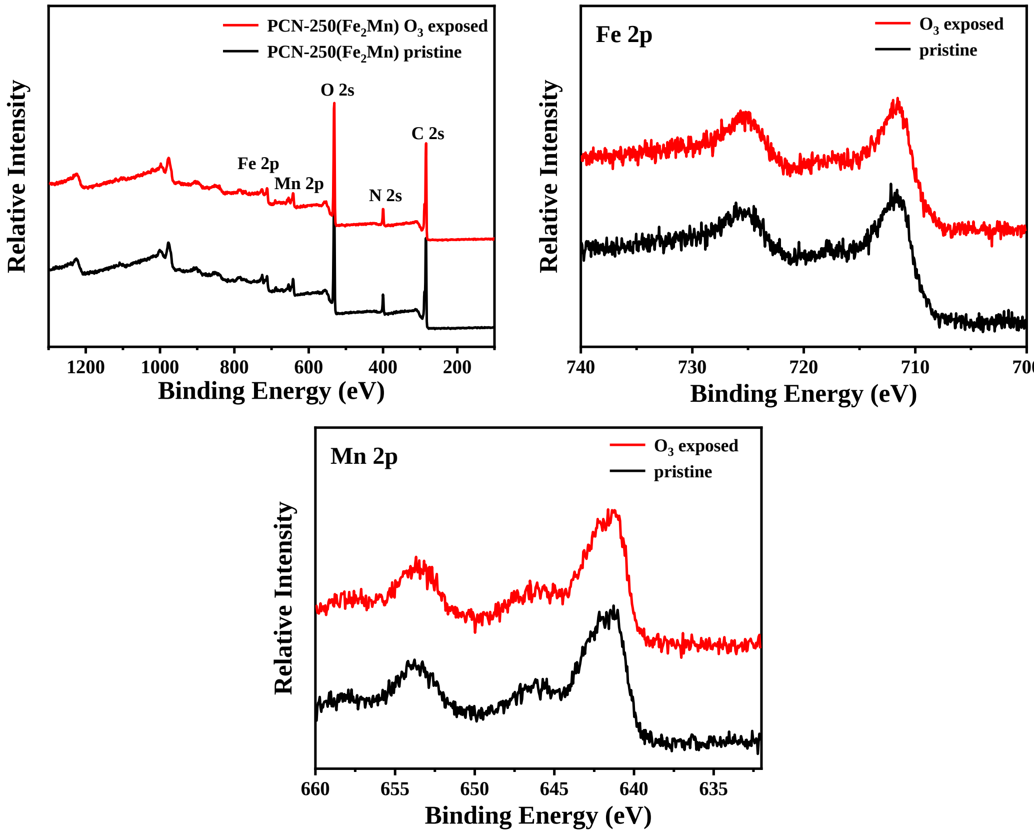

Supplementary Fig. 11. XPS spectra for PCN-250(Fe_2Mn). The XPS spectra of pristine and O_3 exposed PCN-250(Fe_2Mn). The MOF sample was exposed to a continuous O_3-containing humid air flow (RH = 40%; flow rate = 0.5 L min^{-1}; concentration of O_3 = 50 ppm) for 50 hours, and then to a continuous O_3-containing dry air flow (RH < 1%; flow rate = 0.5 L min^{-1}; concentration of O_3 = 50 ppm) for another 50 hours at room temperature.

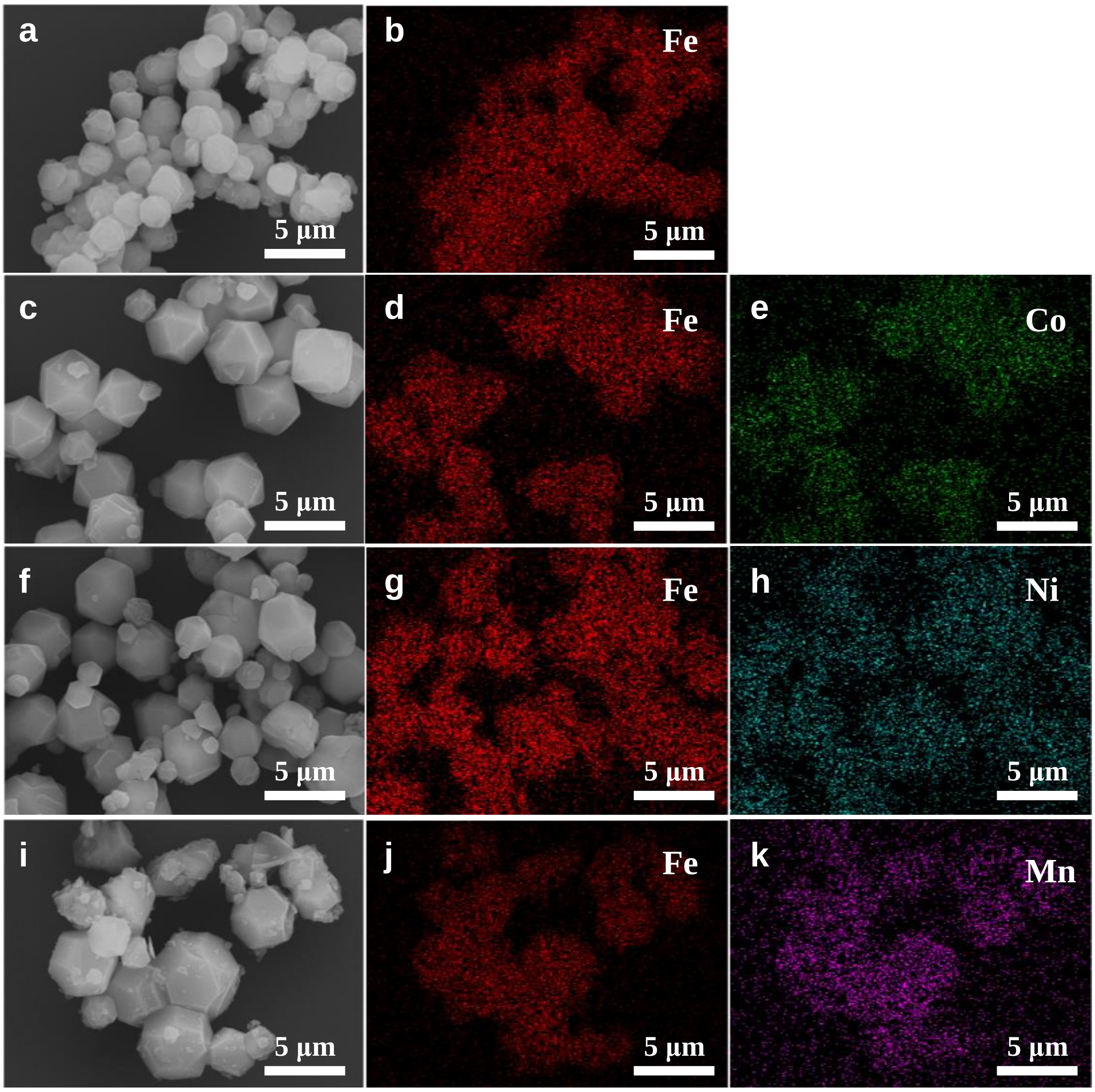

Supplementary Fig. 12. SEM and EDS mapping. The SEM and EDS mapping images for (**a**, **b**) PCN-250(Fe_3), (**c-e**) PCN-250(Fe_2Co), (**f-h**) PCN-250(Fe_2Ni), and (**i-k**) PCN-250(Fe_2Mn).

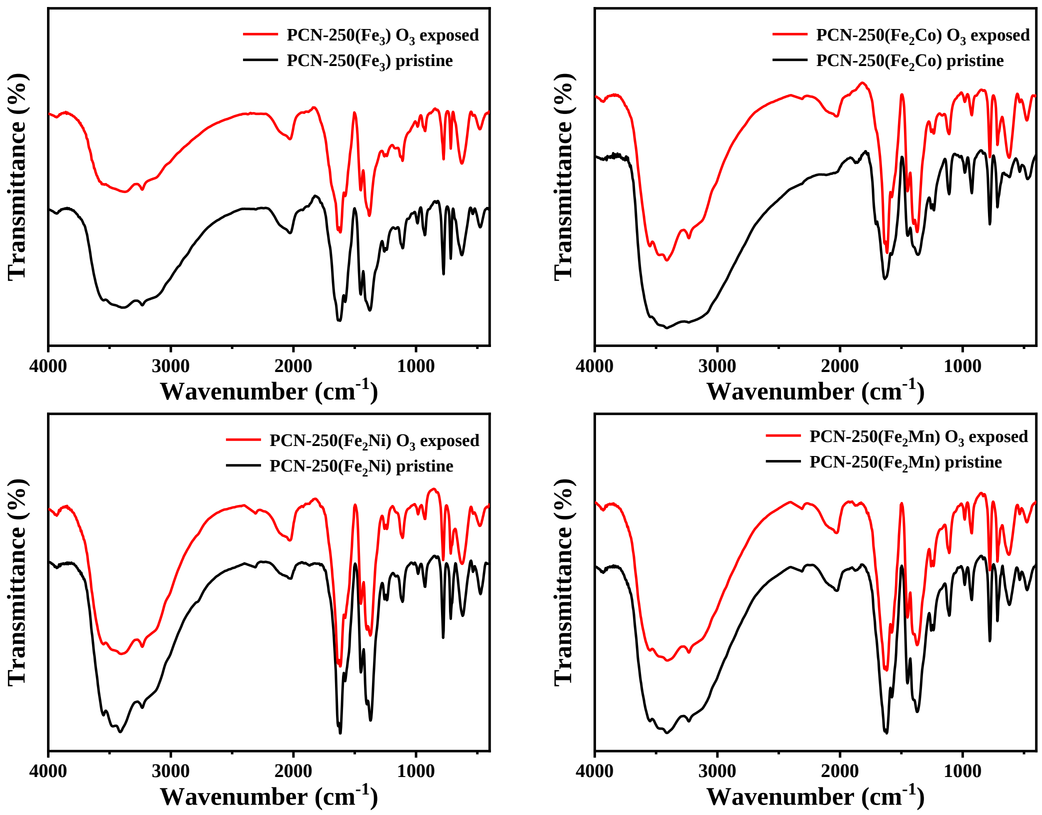

Supplementary Fig. 13. FT-IR spectra. The FT-IR spectra of pristine and O_3 exposed PCN-250 samples. The MOF samples were exposed to a continuous O_3-containing humid air flow (RH = 40%; flow rate = 0.5 L min^{-1}; concentration of O_3 = 50 ppm) for 50 hours, and then to a continuous O_3-containing dry air flow (RH < 1%; flow rate = 0.5 L min^{-1}; concentration of O_3 = 50 ppm) for another 50 hours at room temperature.

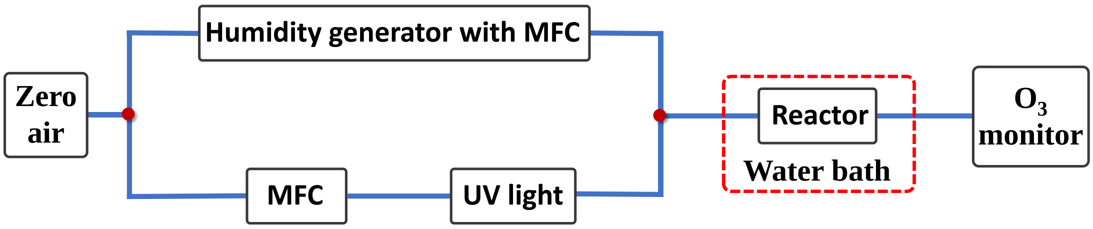

Supplementary Fig. 14. Reactor setup schematic. The schematic of the setup for O_3 decomposition test. The total inlet air flow rate was set to be 1L min^{-1}.

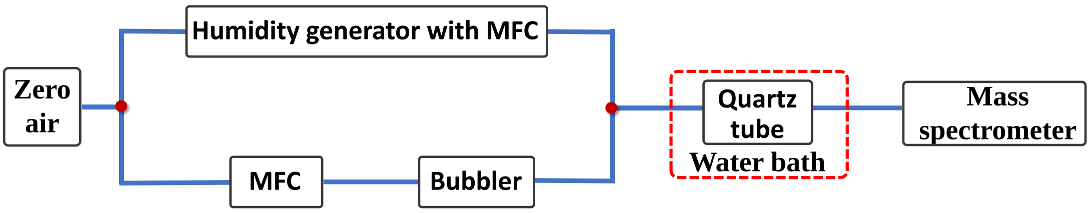

Supplementary Fig. 15. Breakthrough setup schematic. The schematic of the setup for acetone vapor breakthrough experiments. The total inlet air flow rate was set to be 0.2 L min^{-1}, and the tests were carried out at room temperature.

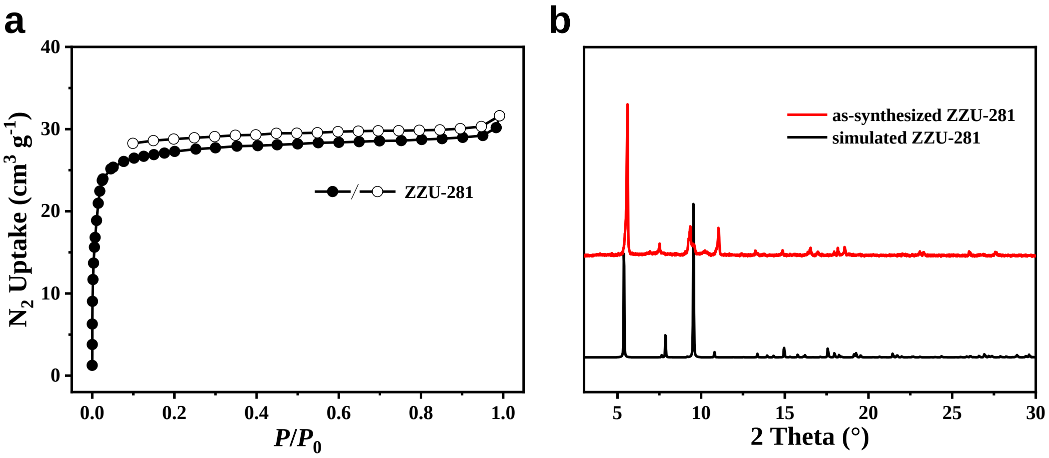

Supplementary Fig. 16. N_2 adsorption and PXRD for ZZU-281. a N_2 adsorption isotherm at 77 K and **b** PXRD pattern of prepared ZZU-281 in comparison with the simulated one.

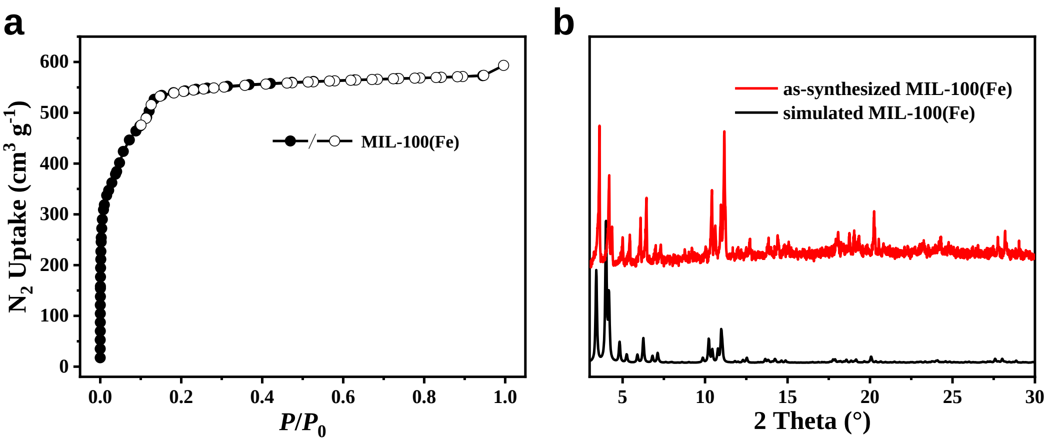

Supplementary Fig. 17. N_2 adsorption and PXRD for MIL-100(Fe). a N_2 adsorption isotherm at 77 K and **b** PXRD pattern of prepared MIL-100(Fe) in comparison with the simulated one.

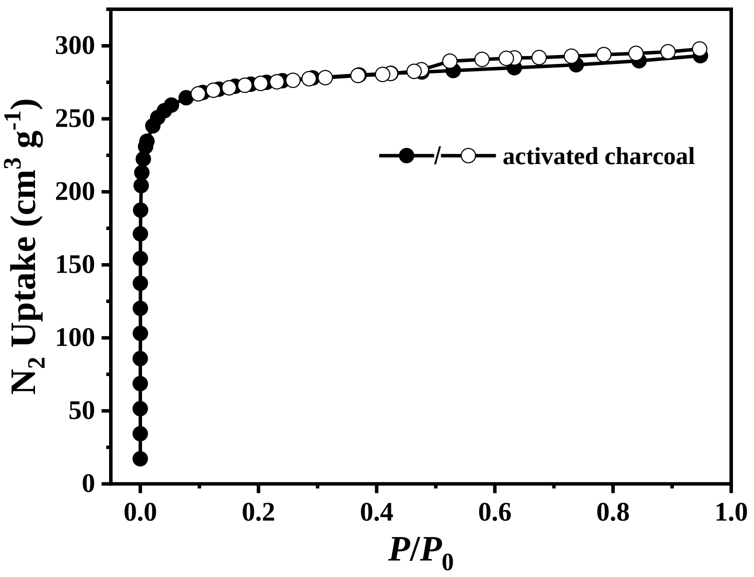

Supplementary Fig. 18. N_2 adsorption for activated charcoal. N_2 adsorption isotherm at 77 K of activated charcoal.

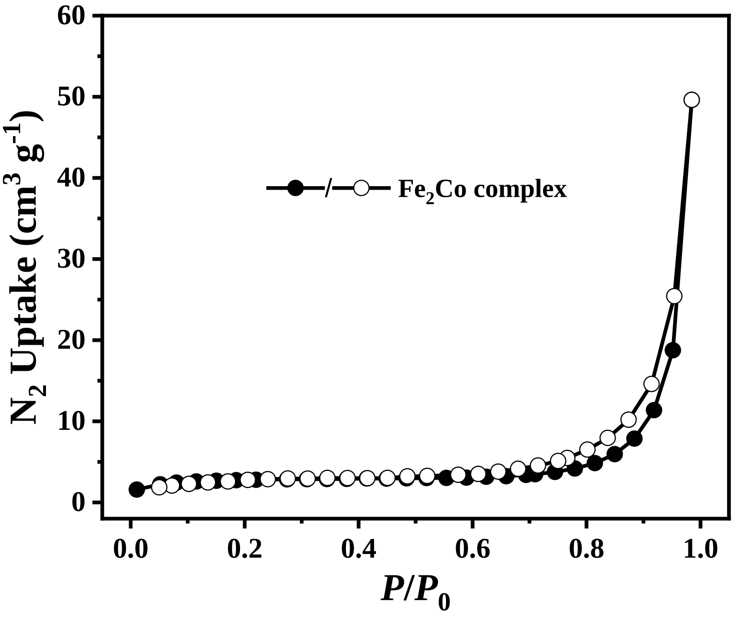

Supplementary Fig. 19. N_2 adsorption for Fe_2Co complex. N_2 adsorption isotherm at 77 K of the Fe_2Co complex.

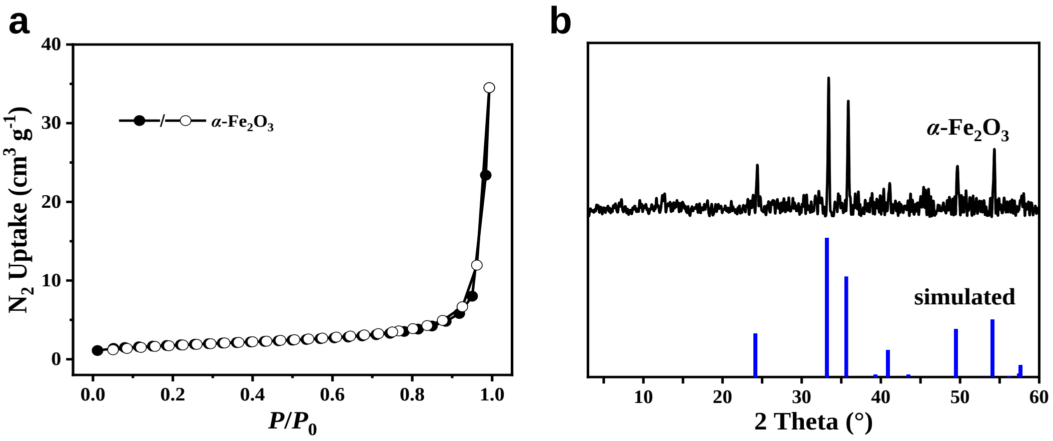

Supplementary Fig. 20. N_2 adsorption and PXRD for α-Fe_2O_3. a N_2 adsorption isotherm at 77 K and **b** PXRD pattern of α-Fe_2O_3 in comparison with the simulated one (JCPDS PDF Card NO.: 86-0550).

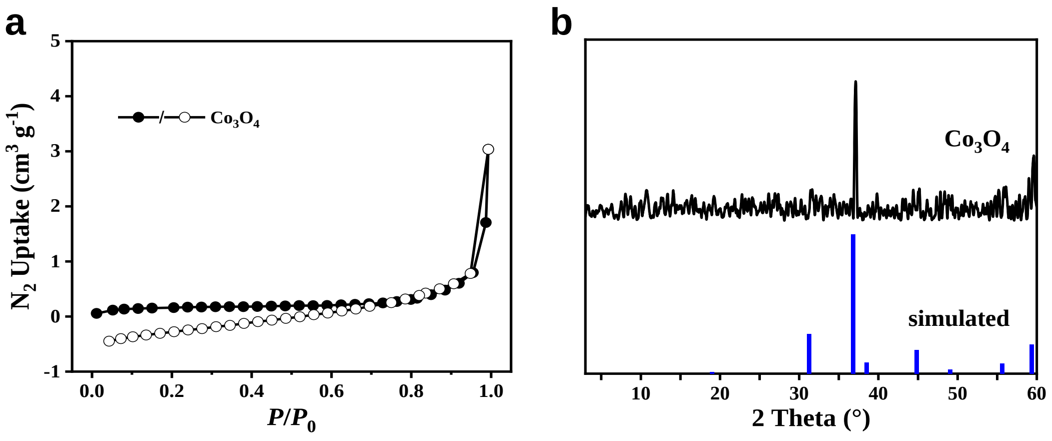

Supplementary Fig. 21. N_2 adsorption and PXRD for Co_3O_4. a N_2 adsorption isotherm at 77 K and **b** PXRD pattern of Co_3O_4 in comparison with the simulated one (JCPDS PDF Card NO.: 78-1970).

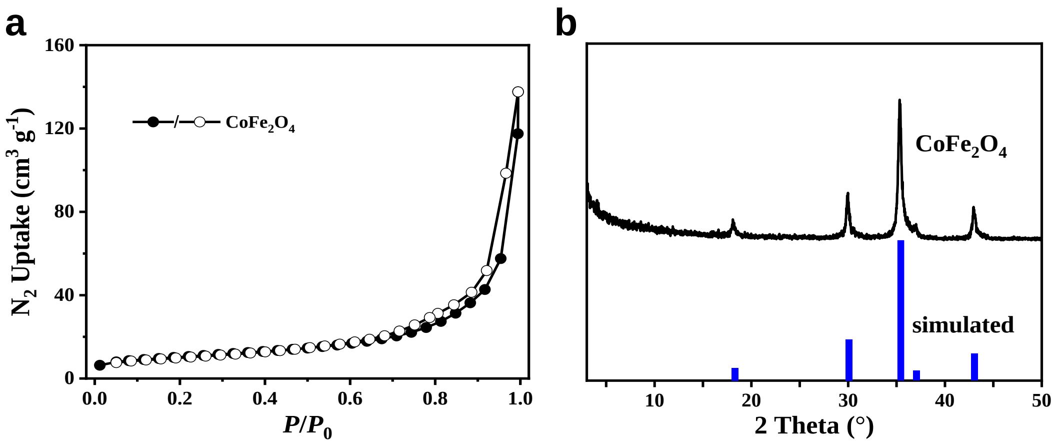

Supplementary Fig. 22. N_2 adsorption and PXRD for $CoFe_2O_4$. a N_2 adsorption isotherm at 77 K and **b** PXRD pattern of $CoFe_2O_4$ in comparison with the simulated one (JCPDS PDF Card NO.: 22-1086).

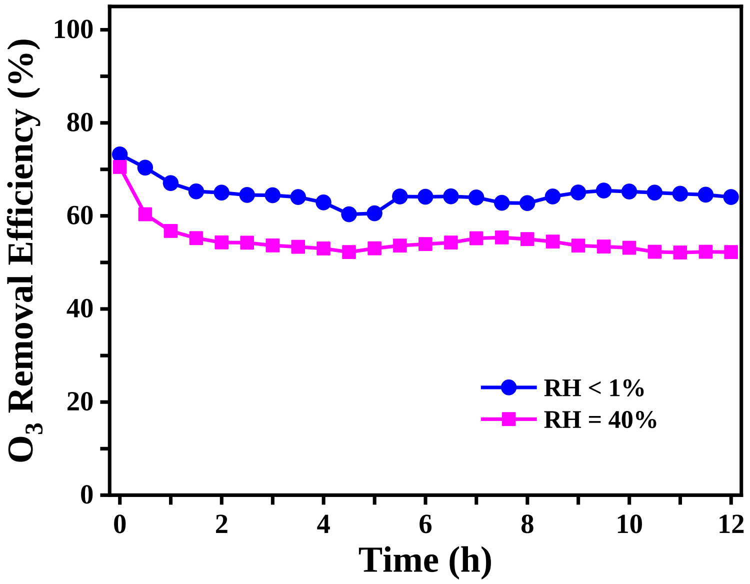

Supplementary Fig. 23. O_3 removal tests for the mixture of Co_3O_4 and *α*-Fe_2O_3. The O_3 removal efficiencies for the physical mixture of Co_3O_4 and *α*-Fe_2O_3 under RH < 1% and RH = 40%, respectively. Other test conditions: 15 mg Co_3O_4 and 15 mg *α*-Fe_2O_3 diluted with 0.2 g quartz sand, concentration of O_3 = 1 ppm, flow rate = 1 L min^{-1}, room temperature.

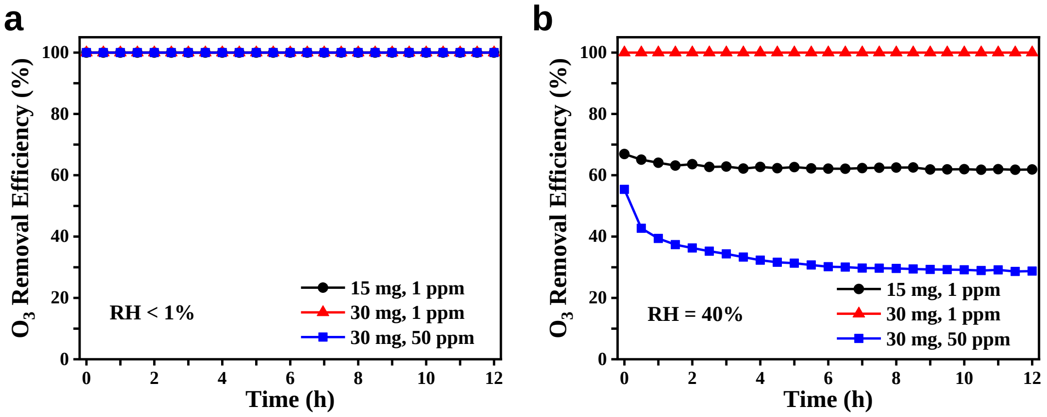

Supplementary Fig. 24. O_3 removal tests for PCN-250(Fe_2Co). The O_3 removal efficiencies for PCN-250(Fe_2Co) when the weight amount or O_3 concentration were changed under **a** RH < 1% and **b** RH = 40%, respectively. Other test conditions: catalyst diluted with 0.2 g quartz sand, flow rate = 1 L min^{-1}, room temperature.

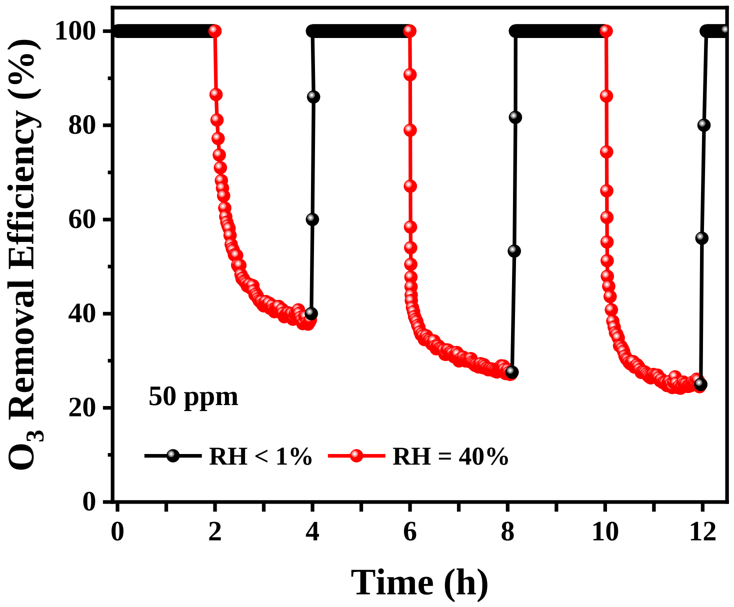

Supplementary Fig. 25. O_3 removal tests for PCN-250(Fe_2Co). The O_3 removal efficiencies for PCN-250(Fe_2Co) at alternate humidity conditions. Other test conditions: 0.03 g catalyst diluted with 0.2 g quartz sand, concentration of O_3 = 50 ppm, flow rate = 1 L min^{-1}, room temperature.

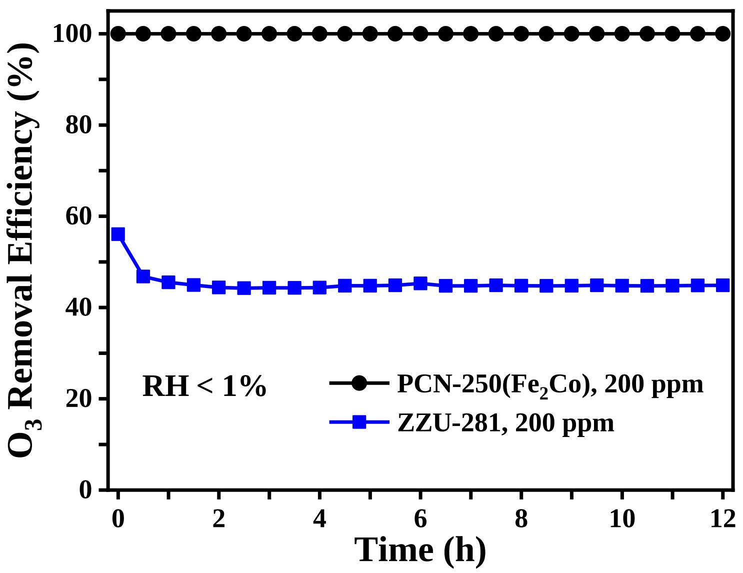

Supplementary Fig. 26. O_3 removal tests for PCN-250(Fe_2Co) and ZZU-281. The O_3 removal efficiencies for PCN-250(Fe_2Co) and ZZU-281 under dry condition. Other test conditions: 0.03 g catalyst diluted with 0.2 g quartz sand, flow rate = 1 L min^{-1}, room temperature.

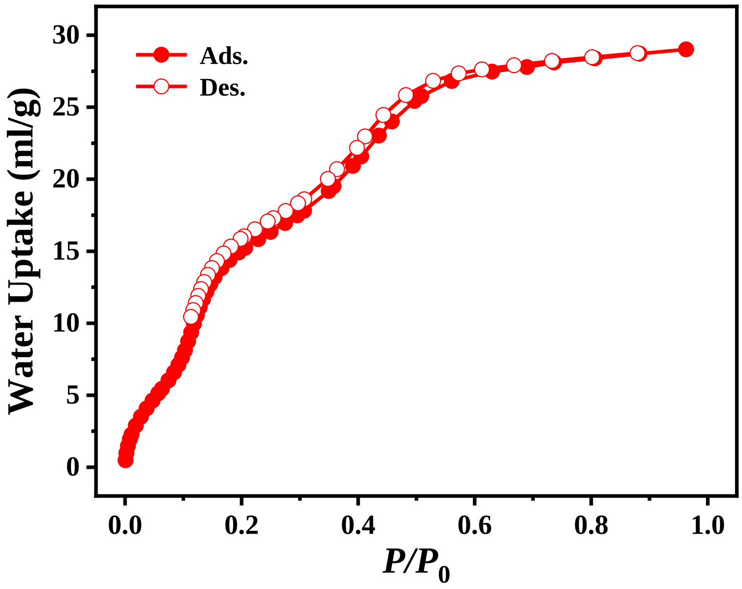

Supplementary Fig. 27. Water adsorption for PCN-250(Fe_2Co). Water adsorption-desorption isotherms of PCN-250(Fe_2Co) recorded at 298 K.

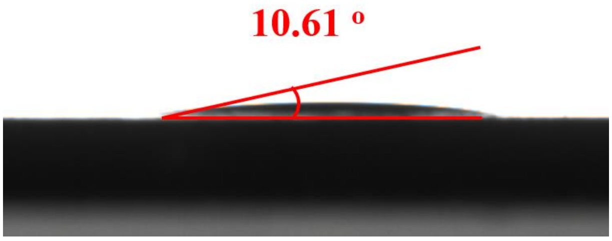

Supplementary Fig. 28. Water contact angle test for PCN-250(Fe_2Co). The static water contact angle of PCN-250(Fe_2Co).

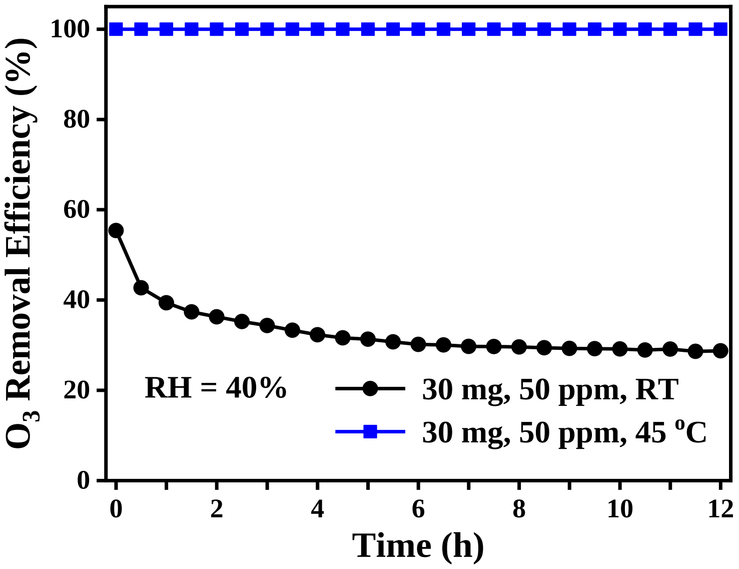

Supplementary Fig. 29. O_3 removal tests for PCN-250(Fe_2Co). The O_3 removal efficiencies for PCN-250(Fe_2Co) at room temperature and 45 °C under humid condition (RH = 40% at room temperature). Other test conditions: 0.03 g catalyst diluted with 0.2 g quartz sand, concentration of O_3 = 50 ppm, flow rate = 1 L min^{-1}.

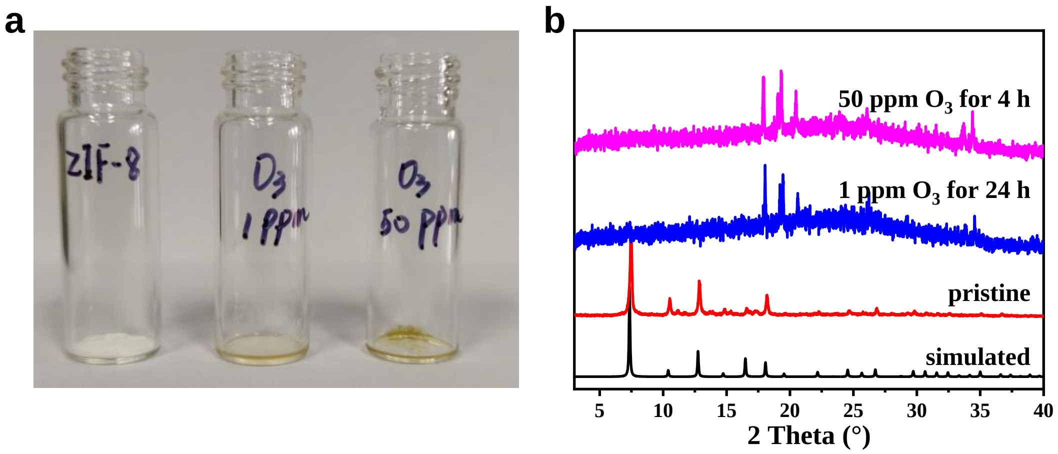

Supplementary Fig. 30. Stability tests of ZIF-8 to O_3. a The photographs and **b** PXRD patterns of simulated, pristine and O_3 exposed ZIF-8 samples. Test conditions: concentration of O_3 = 1 ppm or 50 ppm, RH = 40%, flow rate = 0.5 L min^{-1}, room temperature.

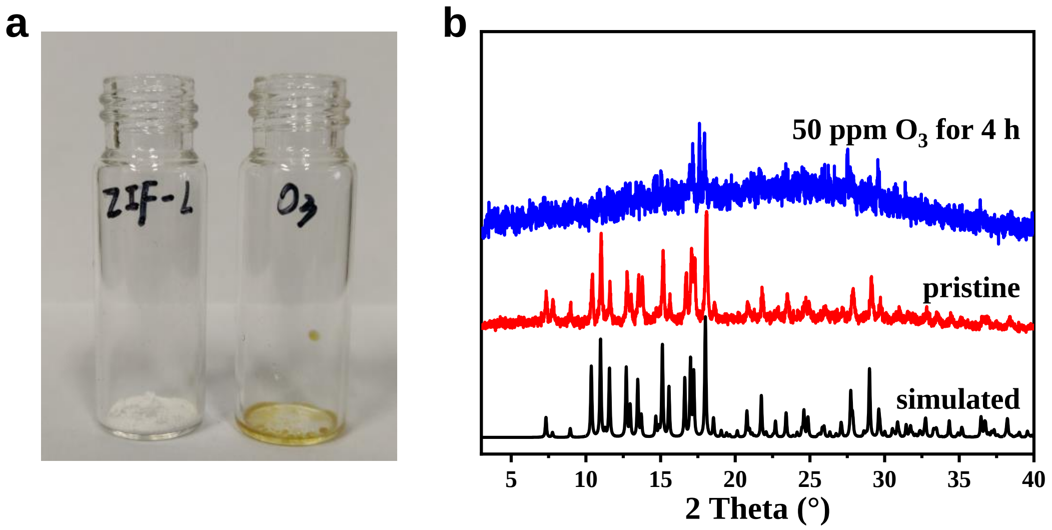

Supplementary Fig. 31. Stability tests of ZIF-L to O_3. a The photographs and **b** PXRD patterns of simulated, pristine and O_3 exposed ZIF-L samples. Test conditions: concentration of O_3 = 50 ppm, RH = 40%, flow rate = 0.5 L min^{-1}, room temperature.

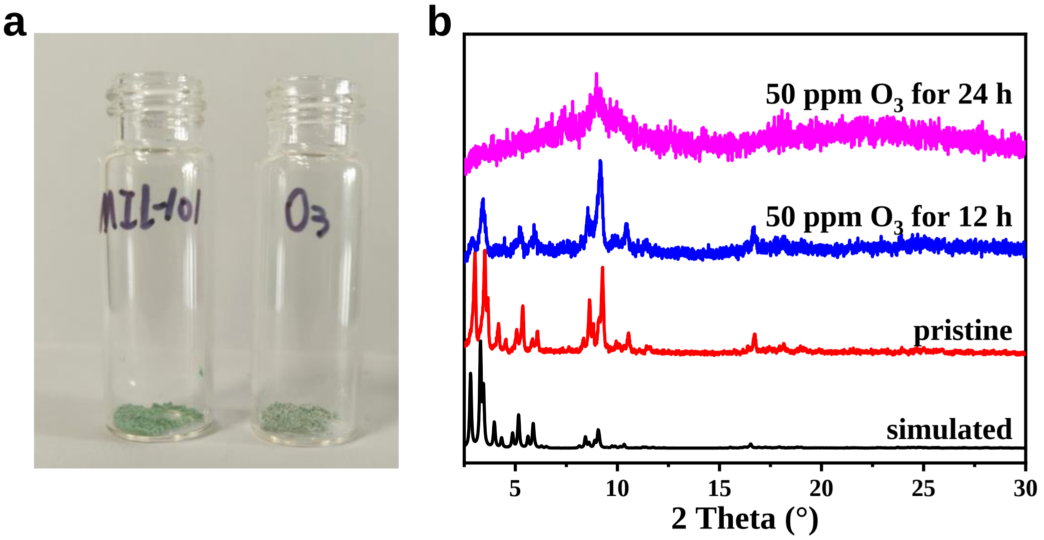

Supplementary Fig. 32. Stability tests of MIL-101(Cr) to O_3. a The photographs and **b** PXRD patterns of simulated, pristine and O_3 exposed MIL-101(Cr) samples. Test conditions: concentration of O_3 = 50 ppm, RH = 40%, flow rate = 0.5 L min^{-1}, room temperature.

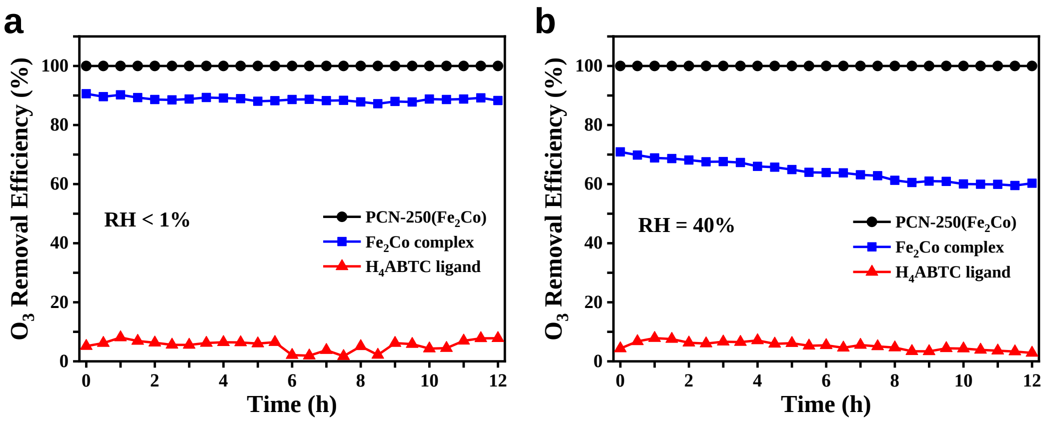

Supplementary Fig. 33. O_3 removal tests for PCN-250(Fe_2Co) and its precursors. The O_3 removal efficiencies for PCN-250(Fe_2Co), [$Fe_2Co(\mu_3\text{-}O)(CH_3COO)_6$], and the H_4ABTC ligand at **a** RH < 1% and **b** RH = 40%, respectively. Other test conditions: 0.02 mmol catalyst diluted with 0.2 g quartz sand, concentration of O_3 = 1 ppm, flow rate = 1 L min^{-1}, room temperature.

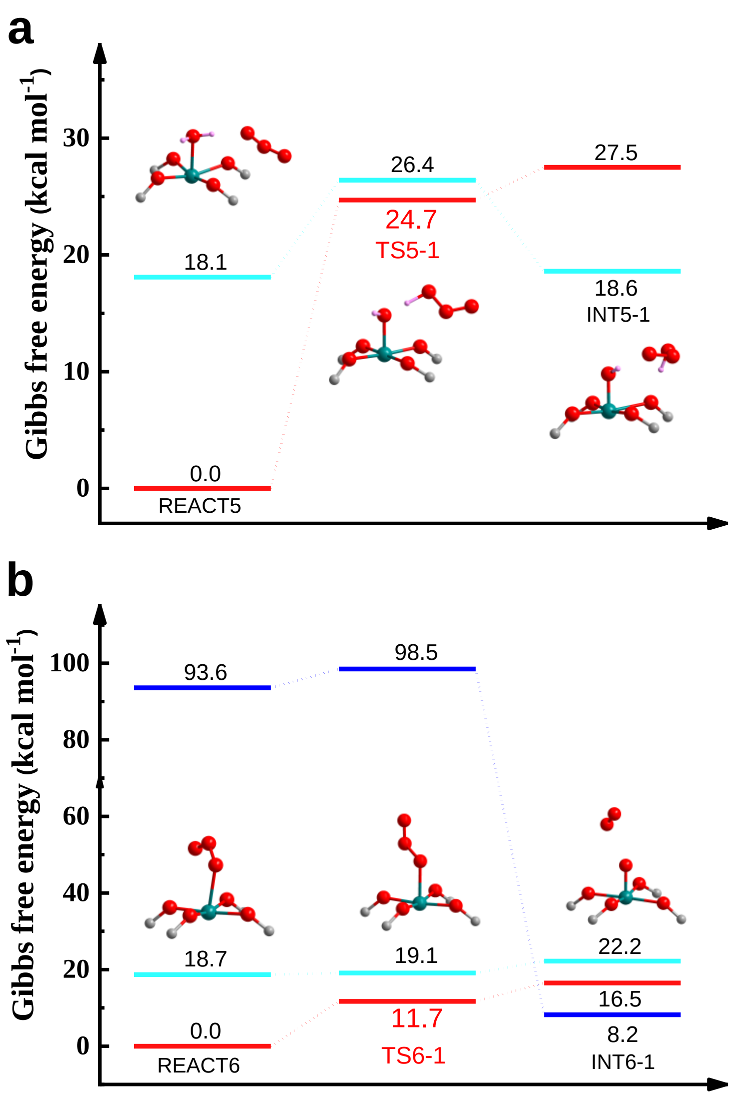

Supplementary Fig. 34. O_3 decomposition mechanism exploration. Optimized structures of stationary points along the first elementary reaction at the Fe(III) site of the PCN-250(Fe_3)-catalyzed O_3 decomposition and corresponding potential energy profile under **a** humid and **b** dry conditions. Red, light blue and dark blue lines denoting the energy levels of the spin state of S = 16, S = 18 and S = 20 of PCN-250(Fe_3) hydroxide complex, respectively. Color code: Fe, turquoise; C, gray; O, red; and H, pink.

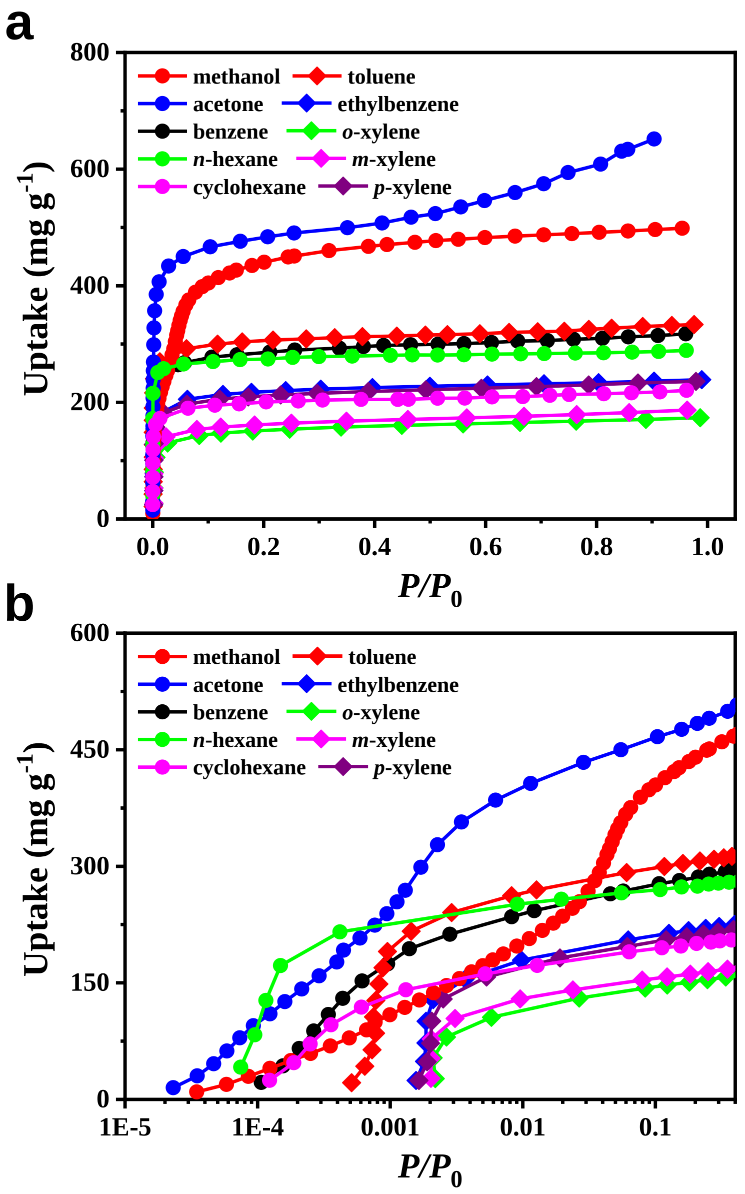

Supplementary Fig. 35. VOCs adsorption studies for PCN-250(Fe$_2$Co). a Vapor adsorption isotherms of PCN-250(Fe$_2$Co) for methanol, acetone, benzene, *n*-hexane, cyclohexane, toluene, ethylbenzene, *o*-xylene, *m*-xylene, and *p*-xylene recorded at 25 °C. **b** Zoom-in view for adsorption data at the low pressure range.

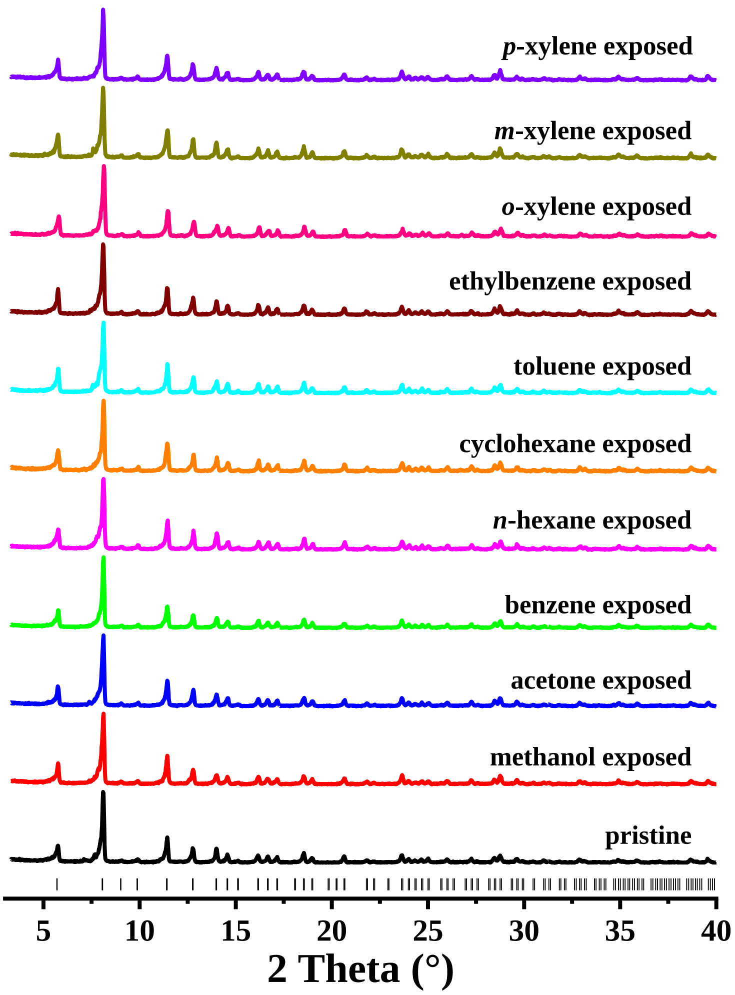

Supplementary Fig. 36. Stability tests of PCN-250(Fe_2Co) to VOCs. PXRD patterns of pristine and VOCs exposed PCN-250(Fe_2Co) samples. The MOF samples were exposed to saturated vapors of different VOCs (methanol, acetone, benzene, *n*-hexane, cyclohexane, toluene, ethylbenzene, *o*-xylene, *m*-xylene, and *p*-xylene) at room temperature for 48 hours, respectively.

Supplementary Table 5. Vapor adsorption data recorded at 25 °C for PCN-250(Fe_2Co).

n-hexane		cyclohexane		benzene	
Absolute Pressure (kPa)	Quantity Adsorbed ($cm^3(STP)g^{-1}$)	Absolute Pressure (kPa)	Quantity Adsorbed ($cm^3(STP)g^{-1}$)	Absolute Pressure (kPa)	Quantity Adsorbed ($cm^3(STP)g^{-1}$)
1.50E-03	10.801	1.60E-03	6.5362	1.36E-03	6.2841
1.91E-03	21.637	2.44E-03	12.604	1.99E-03	12.38
2.32E-03	33.162	3.24E-03	19.027	2.63E-03	18.837
2.99E-03	44.84	4.65E-03	25.62	3.39E-03	25.304
8.39E-03	56.132	7.89E-03	31.661	4.37E-03	31.359
0.1827	65.401	1.71E-02	37.635	5.61E-03	37.341
0.3928	67.085	6.77E-02	43.096	7.85E-03	43.79
1.1145	69.226	0.1672	45.968	1.22E-02	50.112
2.1842	70.31	0.8259	50.645	1.78E-02	55.691
3.1628	71.186	1.4584	52.022	3.60E-02	61.038
4.1727	71.473	2.0269	52.605	0.1055	67.513
5.0687	72.16	2.6587	53.508	0.1558	69.723
6.0156	72.49	3.415	53.98	0.5849	75.964
7.2255	72.773	3.9822	54.392	0.7326	76.991
8.609	73.076	4.8864	54.688	1.3633	79.697
9.4019	73.305	5.7477	54.643	1.9425	80.857
10.317	73.203	5.9989	54.736	2.6972	82.169
11.274	73.332	6.6797	55.197	3.2699	83.192
12.29	73.606	7.3177	55.298	4.3009	84.071
13.329	73.73	7.9585	55.8	4.8523	84.882
14.179	73.859	8.6788	55.953	5.3179	85.436
15.304	74.12	9.3134	56.533	5.9395	85.791
16.328	74.227	9.7668	56.902	6.5713	86.079
17.36	74.519	10.578	57.265	7.1688	86.353
18.321	74.825	11.229	57.673	7.8042	86.923
19.326	75.291	11.897	58.097	8.4074	87.628
18.759	74.88	12.519	58.857	9.0897	87.856
17.861	74.746	12.185	58.707	9.6923	88.523
16.705	74.315	11.561	58.31	10.361	88.959
15.959	74.239	10.912	58.19	10.954	89.678
14.782	73.848	10.25	58.087	11.637	90.31
13.84	73.325	9.5925	57.888	12.277	91.154
12.894	73.326	8.94	57.65	11.982	90.743
11.761	73.291	8.3107	57.432	11.296	90.18
10.973	73.192	7.6594	56.646	10.688	89.706
10.034	72.739	6.9801	56.371	10.069	89.325
8.8586	72.512	6.3815	56.18	9.4371	89.243

7.7642	72.285	5.717	56.144	8.8143	88.806
7.0034	71.843	5.0269	55.905	8.0894	88.341
5.8558	70.868	4.4916	55.314	7.4882	87.989
4.855	70.74	3.7601	54.517	6.9968	87.36
3.8558	70.032	3.1139	53.587	6.2355	87.022
2.8619	69.327	2.5097	52.672	5.6237	86.431
1.8663	68.195	1.8809	52.023	4.9944	86.063
1.0202	66.918			4.2905	85.677
				3.7141	84.883
				3.0953	83.197
				2.4978	82.429
				1.8766	81.221
***o*-xylene**		***m*-xylene**		***p*-xylene**	
**Absolute Pressure (kPa)**	**Quantity Adsorbed (cm^3(STP)g^{-1})**	**Absolute Pressure (kPa)**	**Quantity Adsorbed (cm^3(STP)g^{-1})**	**Absolute Pressure (kPa)**	**Quantity Adsorbed (cm^3(STP)g^{-1})**
2.01E-03	5.6678	2.23E-03	5.7159	1.98E-03	5.1405
1.94E-03	11.304	2.23E-03	11.152	2.28E-03	10.237
2.45E-03	16.95	2.24E-03	16.433	2.42E-03	15.274
5.33E-03	22.348	3.39E-03	22.01	2.47E-03	21.301
2.45E-02	27.541	1.05E-02	27.358	3.01E-03	27.302
7.71E-02	30.259	2.62E-02	29.759	6.39E-03	33.294
0.113	31.098	8.74E-02	32.455	2.29E-02	38.477
0.1658	31.889	0.1348	33.306	7.36E-02	41.602
0.2269	32.563	0.2018	34.086	0.1447	43.406
0.3122	33.284	0.2749	34.737	0.2066	44.215
0.413	33.919	0.3841	35.435	0.2768	44.943
0.5147	34.461	0.5058	36.062	0.3554	45.533
0.609	34.967	0.6226	36.641	0.4663	46.193
0.7022	35.436	0.7362	37.206	0.5905	46.804
0.8177	36.068	0.8405	37.846	0.7109	47.392
0.9079	36.689	0.9445	38.555	0.8302	47.969
0.7349	36.176	1.0595	39.467	0.9431	48.591
0.5936	35.726	0.949	38.819	1.0497	49.302
0.4716	35.277	0.8101	38.334	1.175	49.881
0.3564	34.762	0.6584	37.853	0.9924	49.149
0.2717	34.269	0.5104	37.336	0.8502	48.735
0.1999	33.717	0.4068	36.912	0.6945	48.279
0.1663	33.189	0.3092	36.421	0.5405	47.764
0.111	31.936	0.221	35.834	0.4288	47.32
		0.1461	34.271	0.3247	46.806
				0.232	46.192
				0.151	44.489

acetone		toluene		ethylbenzene	
**Absolute Pressure (kPa)**	**Quantity Adsorbed (cm^3(STP)g^{-1})**	**Absolute Pressure (kPa)**	**Quantity Adsorbed (cm^3(STP)g^{-1})**	**Absolute Pressure (kPa)**	**Quantity Adsorbed (cm^3(STP)g^{-1})**
7.10E-04	5.8246	1.99E-03	5.2166	2.04E-03	5.1641
1.08E-03	11.715	2.50E-03	10.396	2.36E-03	10.326
1.43E-03	17.749	2.84E-03	15.563	2.42E-03	15.364
1.81E-03	24.056	3.02E-03	20.726	2.42E-03	21.275
2.26E-03	30.639	2.91E-03	25.863	2.74E-03	27.109
2.86E-03	36.626	3.03E-03	31.006	4.77E-03	32.709
3.81E-03	42.537	3.19E-03	36.154	1.27E-02	37.859
4.94E-03	48.61	3.43E-03	41.255	8.10E-02	43.387
6.61E-03	54.871	3.70E-03	46.348	0.1645	45.147
8.96E-03	61.431	5.59E-03	52.683	0.2311	45.9
1.22E-02	68.307	1.13E-02	58.547	0.3114	46.54
1.37E-02	74.191	3.20E-02	63.842	0.3937	47.034
1.82E-02	80.158	4.93E-02	65.661	0.5148	47.598
2.36E-02	86.571	0.236	71.093	0.6495	48.117
2.90E-02	92.292	0.4544	72.987	0.7842	48.574
3.47E-02	98.166	0.6275	73.929	0.9168	48.986
4.00E-02	103.92	0.8433	74.721	1.0447	49.395
5.24E-02	115.4	1.076	75.23	1.1747	49.935
6.99E-02	126.56	1.2757	75.686	1.2867	50.474
0.106	137.89	1.4703	76.15	1.0649	49.877
0.1918	148.72	1.711	76.376	0.8757	49.433
0.3527	157.06	1.9119	76.813	0.7034	48.968
0.8824	167.5	2.0658	76.971	0.5776	48.566
1.6905	173.78	2.2938	77.343	0.4653	48.129
3.1866	180.27	2.4988	77.899	0.36	47.631
4.8555	183.94	2.6994	78.197		
6.3747	186.89	2.8861	78.402		
7.8358	189.39	3.0576	79.185		
10.798	192.86	3.2177	79.669		
12.718	196.06	3.4353	80.369		
14.325	199.83	3.6401	80.81		
15.67	202.25	3.7966	81.218		
17.074	206.69	3.5866	80.875		
18.395	210.84	3.407	80.458		
20.084	216.22	3.1842	80.158		
21.67	222.05	2.9802	79.994		
23.025	229.42	2.8007	79.504		
24.827	234.99	2.5489	79.369		
25.998	243.59	2.3426	79.396		

26.33	244.78	2.0092	79.078		
27.796	251.71	1.6525	78.677		
28.976	261.73	1.4135	78.348		
29.142	272.13	1.2285	78.049		
29.209	281.6	1.0326	77.654		
29.228	290.83	0.8528	77.211		
28.938	277.08	0.7607	76.141		
27.957	265.64	0.5569	75.124		
27.389	265.4				
25.943	263.88				
24.432	262.89				
22.965	261.63				
21.485	260.1				
19.786	257.69				
18.432	256.11				
16.741	253.98				
15.255	251.87				
13.697	249.54				
12.201	245.45				
10.573	240.06				
9.2355	236.58				
7.673	232.76				
5.9838	229.01				
4.4929	221.96				
**methanol**					
**Absolute Pressure (kPa)**	**Quantity Adsorbed (cm^3(STP)g^{-1})**	**Absolute Pressure (kPa)**	**Quantity Adsorbed (cm^3(STP)g^{-1})**	**Absolute Pressure (kPa)**	**Quantity Adsorbed (cm^3(STP)g^{-1})**
5.50E-04	6.7497	0.4226	178.02	7.4772	332.13
9.21E-04	13.492	0.4918	187.57	8.0768	334.06
1.35E-03	20.695	0.5519	196.88	8.7162	335.65
1.96E-03	27.956	0.598	204.25	9.4727	337.84
2.82E-03	34.92	0.6433	212.92	10.334	339.48
3.97E-03	41.427	0.682	220.47	11.15	341.03
5.60E-03	48.163	0.7122	225.84	11.953	342.53
7.78E-03	55.351	0.7481	232.15	12.731	344.09
1.05E-02	62.592	0.7853	238.36	13.554	345.71
1.22E-02	69.638	0.8222	243.7	14.33	347.45
1.57E-02	76.221	0.867	249.33	15.1	349.02
2.04E-02	82.913	0.9453	256.82	14.085	347.51
2.63E-02	89.478	1.0296	262.78	13.342	346.27
3.36E-02	95.984	1.2195	272.16	12.549	345.1
4.21E-02	102.55	1.4128	278.84	11.775	343.86

5.27E-02	108.8	1.5896	283.24	10.975	342.69
6.51E-02	114.83	1.8686	289.69	10.28	340.67
7.90E-02	120.49	2.1916	295.31	9.4191	338.81
9.41E-02	125.68	2.3844	298.7	8.5795	336.18
0.113	131.04	2.8325	304.28	7.8304	334.09
0.1425	138.12	3.1797	308.24	7.0509	330.43
0.1774	144.83	3.8634	314.52	6.3275	327.7
0.2222	152.19	4.0383	315.72	5.4656	323.65
0.2686	158.78	5.0276	322.06	4.7128	320.49
0.3163	164.99	6.1497	327.16		
0.3751	172.12	6.6822	329.46		

Supplementary References

1 Canioni, R. *et al.* Stable polyoxometalate insertion within the mesoporous metal organic framework MIL-100(Fe). *J. Mater. Chem.* **21,** 1226-1233 (2011).

2 Sun, Z.-B., Si, Y.-N., Zhao, S.-N., Wang, Q.-Y. & Zang, S.-Q. Ozone Decomposition by a Manganese-Organic Framework over the Entire Humidity Range. *J. Am. Chem. Soc.* **143,** 5150-5157 (2021).

3 Férey, G., Mellot-Draznieks, C., C. Serre, F. M., J. Dutour, S. S. & Margiolaki, I. A Chromium Terephthalate–Based Solid with Unusually Large Pore Volumes and Surface Area. *Science* **309,** 2040-2042 (2005).

4 Pan, Y., Liu, Y., Zeng, G., Zhao, L. & Lai, Z. Rapid synthesis of zeolitic imidazolate framework-8 (ZIF-8) nanocrystals in an aqueous system. *Chem. Commun.* **47,** 2071-2073 (2011).

5 Chen, R. *et al.* A two-dimensional zeolitic imidazolate framework with a cushion-shaped cavity for CO_2 adsorption. *Chem. Commun.* **49,** 9500-9502 (2013).
